# Recent Advances in the Synthesis and Application of Polymer Compartments for Catalysis

**DOI:** 10.3390/polym12102190

**Published:** 2020-09-24

**Authors:** Tai-Lam Nghiem, Deniz Coban, Stefanie Tjaberings, André H. Gröschel

**Affiliations:** Physical Chemistry and Centre for Soft Nanoscience (SoN), University of Münster, 48149 Münster, Germany; tnghiem@uni-muenster.de (T.-L.N.); dcoban@uni-muenster.de (D.C.); stefanie.tjaberings@uni-muenster.de (S.T.)

**Keywords:** block copolymers, cascade reactions, catalysis, controlled polymerization techniques, nanostructures, organocatalysis, polymer architectures, self-assembly, transition metal catalysis

## Abstract

Catalysis is one of the most important processes in nature, science, and technology, that enables the energy efficient synthesis of essential organic compounds, pharmaceutically active substances, and molecular energy sources. In nature, catalytic reactions typically occur in aqueous environments involving multiple catalytic sites. To prevent the deactivation of catalysts in water or avoid unwanted cross-reactions, catalysts are often site-isolated in nanopockets or separately stored in compartments. These concepts have inspired the design of a range of synthetic nanoreactors that allow otherwise unfeasible catalytic reactions in aqueous environments. Since the field of nanoreactors is evolving rapidly, we here summarize—from a personal perspective—prominent and recent examples for polymer nanoreactors with emphasis on their synthesis and their ability to catalyze reactions in dispersion. Examples comprise the incorporation of catalytic sites into hydrophobic nanodomains of single chain polymer nanoparticles, molecular polymer nanoparticles, and block copolymer micelles and vesicles. We focus on catalytic reactions mediated by transition metal and organocatalysts, and the separate storage of multiple catalysts for one-pot cascade reactions. Efforts devoted to the field of nanoreactors are relevant for catalytic chemistry and nanotechnology, as well as the synthesis of pharmaceutical and natural compounds. Optimized nanoreactors will aid in the development of more potent catalytic systems for green and fast reaction sequences contributing to sustainable chemistry by reducing waste of solvents, reagents, and energy.

## 1. Introduction

**Figure 1:**
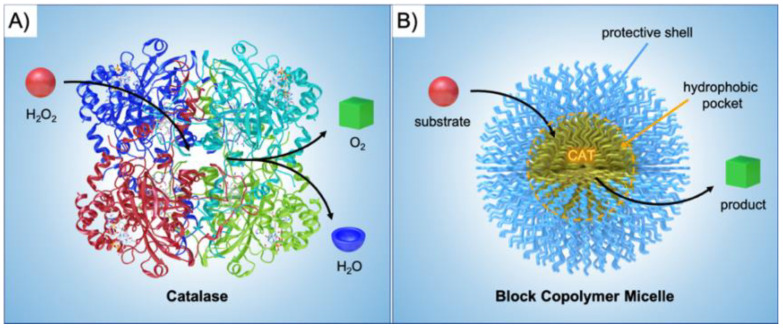
Catalysis in compartments. (**A**) The enzyme catalase converting H_2_O_2_ to water and oxygen. (**B**) Block copolymer micelle with catalyst in the hydrophobic core compartment.

Catalysts have become indispensable components in almost every branch of synthetic chemistry and industry, as they alleviate reaction conditions, can steer reaction paths, and enhance product selectivity [1]. Since catalysis is without a doubt a key technology that is essential to tackle current global challenges (e.g., mobile energy, pharmaceuticals, clean water and air), it is not surprising that a major part of scientific research is devoted to developing more potent catalyst systems. Nature has already developed a multitude of biological nanoreactors (e.g., enzymes, organelles) that are able to synthesize complex organic compounds through highly sophisticated catalytic reaction cycles [2]. Biological nanoreactors are self-assembled or self-organized from structural elements that are equipped with functionalities to fulfill catalytic tasks within the final nanostructure. As a prime example, enzymes display extraordinary catalytic abilities with turn over frequencies (TOFs) unmatched by synthetic catalysts in most cases. Catalase, for instance, is an immensely potent catalyst able to convert H_2_O_2_ into O_2_ and H_2_O [3] as fast as the substrate can reach the reactive center leading to reaction rates close to the diffusion limit (Figure 1A). A common scheme for biological nanoreactors is the incorporation of catalysts in pockets to protect the active site from cross-reactions or harmful reagents, and to channel the substrate towards the catalyst and products away from it. Inspired by these properties, chemists and materials scientists have invested immense efforts to create building blocks for self-assembled nanostructures with advanced functionality and precise composition [4,5,6,7,8,9,10]. Synthetic chemistry has access to a broad range of materials properties to mimic biological nanoreactors on a basic level, but also to devise entirely new systems that are more robust, responsive, and tailored to application needs [11,12,13,14,15]. The basic idea remains the same: design nanoreactors that safely store the catalytic center within a compartment, while allowing substrates to enter and participate in the reaction (Figure 1B).

In homogenous catalysis, catalysts freely diffuse in the reaction mixture, which typically leads to high reaction rates due to fast and easy access of the active centers [16]. After the reaction is completed, however, separation of catalysts from the product can be problematic (e.g., requiring solvent extraction) or they cannot be removed at all. Since catalysts are usually the most expensive component of the reaction or may contain harmful elements, recovery and reuse is generally desired. One way to facilitate separation of catalysts from the product is their immobilization on larger nano- or microparticles that can be removed by filtration or centrifugation. Although the reduced diffusion coefficient of particles (as compared to molecules) may negatively affect the reaction rate, heterogeneous catalysts have proven to be very beneficial. Another possibility is to encapsulate catalysts into compartments, e.g., in polymer vesicles (polymersomes) or microcapsules, to create either a heterogeneous or a heterogenized homogeneous catalyst system that allows substrates to diffuse to the active site, while the catalyst/microcapsule can likewise be removed for reuse by centrifugation or filtration. There are some additional benefits to confining the catalyst into nanocompartments. Hydrophobic pockets shield the catalyst from potentially harmful chemicals, opening routes, e.g., to conducted catalysis in water with water-susceptible catalysts. The confining nanoenvironment can also enhance the reaction rate especially for hydrophobic substrates in water, because both catalyst and substrate are localized with high concentrations in the hydrophobic compartment (concentrator effect) [17,18,19].

**Figure 2:**
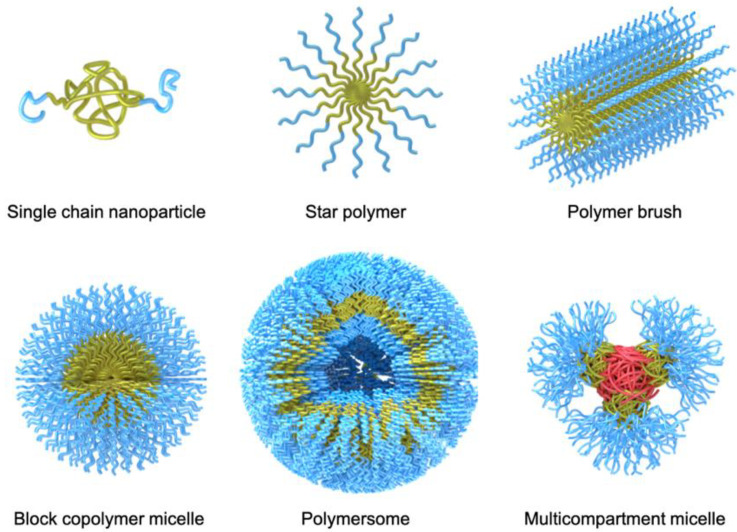
Selection of frequently used polymer compartments for catalysis.

Over the years, a large variety of compartmentalized nanomaterials have been developed for catalysis. Polymer chemistry has access to a diverse toolbox of synthetic methods for the formation of polymer nanostructures. With the advancement of controlled/”living” polymerization techniques [20] including ionic polymerization, reversible addition-fragmentation chain transfer (RAFT) polymerization, atom transfer radical polymerization (ATRP), nitroxide-mediated polymerization (NMP), and ring-opening anionic, cationic, and metathesis polymerization (AROP, CROP, and ROMP), complex polymer architectures become more and more accessible (Figure 2). Aside from the established synthesis of amphiphilic block copolymer (BCPs) that are useful for self-assembly into BCP micelles, polymersomes [14], and multicompartment micelles [21], the synthesis of molecular polymer nanoparticles, such as star polymers [22] and cylindrical polymer brushes [23], has also become more state-of-the-art. Recently, molecular engineering along the polymer chain provided supramolecular binding sites to induce intramolecular chain segment association into single chain polymer nanoparticles [24]. In this review, we will highlight some examples for each of these polymer nanostructures, where one or more catalysts are hosted and employed in diverse reactions. There are of course a range of other polymer scaffolds that have demonstrated high potential in catalytic reactions, e.g., dendrimers [25], microbeads (resins or cross-linked microparticles, microgels) [26,27], and metal-organic frameworks [28]. Throughout this review, we will, however, focus on polymer nanostructures synthesized mainly with controlled polymerization techniques (with few intriguing exceptions).

**Figure 3:**
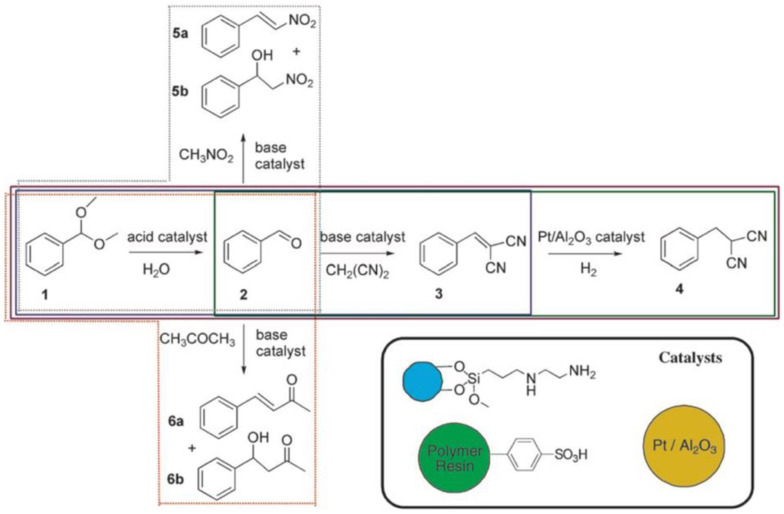
Multistep cascade reaction mediated by a combination of three different supported catalysts in a one-pot reaction. Each colored box shows a different reaction path. Reproduced with permission of [50]. Copyright 2006 Wiley-VCH.

The second component is of course the catalytic site that needs to be incorporated within the polymer compartment. This can be done either supramolecularly through compatibilization (e.g., solubilization) or covalently through copolymerization of properly modified catalyst monomers. Successful examples for heterogeneous catalysis using polymer materials include transition metal catalyzed Heck coupling [29], Suzuki-Miyaura coupling [30], hydroxycarboxylation [31], and hydroformylation [32], but also organocatalytic reactions such as acidic hydrolysis, Knoevenagel condensation, Henry reaction, and asymmetric aldol reactions (these reactions will reappear throughout the review). Given that there is a much larger number of highly potent transition metal and organocatalysts [33], examples for catalytic reactions in polymer compartments are expected to increase over the years. As mentioned above, the site isolation of catalysts in compartments opens the opportunity to accommodate multiple catalysts in the same space without mutual interference. Compartmentalization is essential to perform multiple catalytic steps without interference of reactants and/or catalysts [34]. In cascade catalysis, often called domino or tandem catalysis, two or more catalytic reactions occur consecutively and the starting reagent traverses through at least one intermediate product prior to the formation of the end product. By strict definition of the term, the reaction conditions should not change during the reaction cascade and transformations proceed without addition of further reagents [35]. Due to the sequential transformation as opposed to individual reactions, multistep reactions can be performed in one pot. Multistep one-pot reactions are in high demand (e.g., for the pharmaceutical industry), as they are atom efficient and require considerably less amounts of solvent (no extraction of intermediates), adsorbents, reagents, and energy, especially when conducted in bulk or water under mild conditions [36,37,38,39,40,41]. However, catalysts often interfere with each other or even annihilate each other (e.g., acid and base catalysts). The strict spatial separation of these “wolf and lamb” catalysts [42] is an absolute requirement to prevent irreversible quenching. At the same time, the distance of reactive sites should be minimized (few nanometers) to reduce diffusion paths and reaction time, increase overall yield, and reach high enantiomeric purity (e.g., by rapid conversion of unstable intermediates). The concept of polymer compartmentalization can therefore aid in the development of sequential catalytic reaction platforms. While tandem catalysis is occurring rather frequently in nature, such as biocatalytic multistep substrate conversions mediated by multiple enzymes in cells, the fabrication of synthetic systems for sequential catalysis is still an ongoing development with high research interest [43]. Within the last decade, tremendous progress has been made in the development of polymeric systems for cascade reactions exploiting the combination of catalyst types (metal, organic, enzymatic) with polymer supports/scaffolds (star polymers, BCP micelles, polymersomes, and nanoparticles) for the targeted synthesis of complex small organic compounds with defined chemo-, stereo-, and regioselectivity [44,45]. Although this review focuses on polymer compartments, there are other compelling catalytic systems that should not go unmentioned, e.g., the immobilization of catalysts on high surface area inorganic materials such as metal-organic frameworks (MOFs), zeolites, alumina, or silica [46,47,48]. They were shown, for instance, to embed a polymeric acid catalyst and a small organic base catalyst in two separated matrices to avoid their mutual annihilation, thereby enabling a two-step sequential reaction [49]. In one case, perfluorinated Nafion or molybdic acid was enclosed in silica, while the guanidine base catalyst, 1,5,7-triazabicylco[4.4.0]dec-5-ene, was covalently attached to a sol-gel material known as Ormosil. While the acid catalyzed the rearrangement reaction of 2,3-dimethylbutane-2,3-diol into 2,2-dimethylbutan-3-one, the resulting ketone was converted by the base catalyst to a dinitrile product with malononitrile in a condensation reaction. Such sol-gel materials provide a convenient route for one-pot reactions and are one of many solutions to the “wolf and lamb” dilemma in catalysis. Related are mixtures of colloids that provide site isolation of the catalyst as exemplified on the three-step one-pot cascade sequence employing three different supported catalysts (Figure 3) [50]. Magnetite ferrite particles were modified with primary amine base catalysts *via* silane chemistry, a polymer resin was modified with *p*-phenylsulfonic acid, and the metal catalyst, Pt/Al_2_O_3_, was embedded within a membrane. This system was able to perform the acid-catalyzed deacetalization of benzaldehyde dimethyl acetal followed by a base-catalyzed Knoevenagel reaction with malonitrile to benzylidenemalononitrile, and finally, the hydrogenation to benzylmalononitrile through Pt. The close proximity of multiple catalysts was particularly important for this example in order to suppress the formation of unwanted side products as illustrated in the colored boxes in Figure 3. The individual catalysts could be recovered gravimetrically and magnetically and were reused for the same reaction without any noticeable decline in catalytic activity. One of the aims to improve multi-step one-pot reactions is to combine as many catalysts into one nanoreactor or pot as possible, while avoiding cross-reactions between substrates, intermediates, and by-products.

For this review, we clustered polymer-supported catalytic reactions according to the used polymer compartments, i.e., we first discuss single chain polymer nanoreactors resembling the smallest compartment, then we give some examples for molecularly designed polymer nanoparticles (star and cylindrical brushes), before we proceed to self-assembled BCP micelles and polymersomes. In this context, we will also introduce promising combinations of different catalysts and polymer systems resulting in diverse nanoreactor systems. We close this review by giving some of our future views and perspectives on the highly active field of polymer nanoreactors.

## 2. Single Chain Polymer Nanoparticles

Enzymes and proteins are complex 3D structures of precisely sequence-defined and folded biopolymers. Although synthetic analogues are currently beyond our reach, mimicking the nanopockets of enzymes that contain the active site has always inspired polymer chemists to create functional chains able to “fold” into unimolecular or single chain polymer nanoparticles (SCPNs) with a catalytic nanopocket [51]. Over the years, several concepts emerged to introduce binding sites at specific locations along the polymer chain that lead to a predictable association of chain segments into SCPNs [24]. The catalytic site is thereby enveloped in the hydrophobic pocket by one part of the chain, while water-soluble segments provide stabilization of the SCPN in aqueous environment. Following this concept, a range of SCPNs were designed as catalytic nanoreactors [52,53] that provide nanoconfined environments with special properties [54].

**Figure 4:**
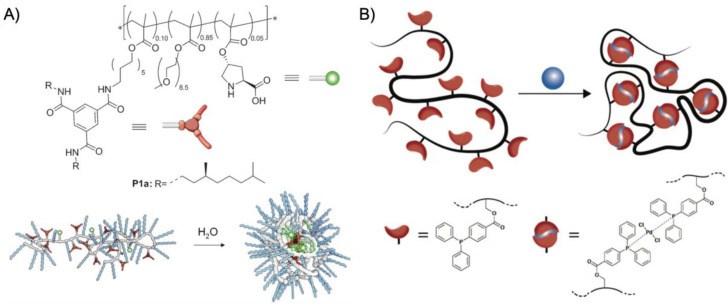
Example for single chain polymer nanoparticles (SCPN) catalysis. (**A**) SCPN with *L*-proline for the enantioselective aldol reaction of cyclohexanone and *p*-nitrobenzaldehyde. (**B**) Selective point folding of SCPNs promoted by triphenylphospane coordination with dichloro(1,5-cyclooctadiene)palladium. (**A**) reproduced with permission of [58]. Copyright 2013 Wiley-VCH. (**B**) reproduced with permission of [63]. Copyright 2015 Royal Society of Chemistry.

A first example for an SCPN catalyst was given by supramolecular chain folding promoted by H-bonding benzene-1,3,5-tricarboxamide (BTA) [55]. Copolymers of BTA methacrylate (BTAMA) and oligoethylene glycol methacrylate (OEGMA, *M*_n_ = 500 g·mol^−1^) were first synthesized controlled living radical polymerization (CRP) using chloro(indenyl)bis(triphenyl-phosphine)ruthenium(II) (Ru(ind)Cl(PPh_3_)_2_) as catalyst. The 4-(Diphenylphosphino)styrene (DPS) ligands were added at about 50% conversion and immediately copolymerized to full consumption leading to a final copolymer sequence of P(OEGMA-*co*-BTAMA)-*b*-P(OEGMA-*co*-BTAMA-*co*-DPS)-*b*-P(OEGMA-*co*-BTAMA) with *M*_n_ = 35.2 kg·mol^−1^ and *M*_w_/*M*_n_ ≈ 1.5. The DPA ligands coordinated RuCl_2_(PPh_3_)_3_ with about 50 µmol·g^−1^ Ru/polymer. The combination of Rh-coordination and hydrogen-bonding-driven association of BTA units facilitated intramolecular chain folding into unimolecular nanoparticles with *R*_h_ ~ 3–4 nm, stabilized in water (or DMF) by the terminal, OEGMA-rich chain ends. The SCPN nanoreactor was employed in the Ru-catalyzed transfer hydrogenation of cyclohexanone to cyclohexanol with sodium formate as the hydrogen source at 40 °C, leading to quantitative conversion after 18 h with (cyclohexanone)/(Ru) = 200/1 (86% conversion for acetophenone). The SCPN provided a hydrophobic cavity that promoted the transfer hydrogenation without decomposition of the polymer. To better understand the structuring role of BTA groups on the catalytic activity, BTAMA was replaced with lauryl methacrylate (LMA) as the hydrophobic component [56]. RAFT polymerization of polymers with BTAMA or LMA led to comparable *M*_n_ = 30 kg·mol^−1^ and *M*_w_/*M*_n_ = 1.36. Coordination with Ru and self-assembly to SCPN resulted in comparable *R*_h_ of 4.4. and 5.8 nm, confirming that BTA is not essential for the stabilization of the hydrophobic nanopocket. The catalytic activity was also found to be the same for both SCPNs, giving TOF of 4.4 h^−1^ and 4.3 h^−1^, demonstrating that efficient catalysis indeed required the hydrophobic pocket but was independent of the BTA or LMA environment. However, the LMA compartment was able to catalyze more hydrophobic substrates such as 4-methyl-, 4-ethyl-, and 4-propyl-cyclohexanone. By addition of *tert*-butylhydroperoxide in 5-fold excess to cyclohexanol, oxidation back to cyclohexanone was realized in water at rt reaching 93% conversion in 13 min [57]. The nanopocket of the SCPN displayed selectivity for more apolar substrates as exemplified on a mixture of 4-tetrahydropyranol, cyclohexanol, and 4-*tert*-butylcyclohexanol. Whereas the most apolar 4-*tert*-butylcyclohexanol reached > 99% conversion in 4 min, the less apolar cyclohexanol reached 70% in the same time, and the least apolar 4-tetrahydropyranol merely 20%. This verified that the polarity between substrate and hydrophobic nanopocket should match so that substrates enrich in the nanoreactor instead of the continuous phase. Building on the SCPNs of P(OEGMA-*co*-BTAMA), *L*-proline was attached to the polymer chain instead of DPS to give P(OEGMA-*co*-BTAMA-*co*-*L*-ProMA) (Figure 4A) [58]. *L*-proline is a prominent organocatalyst in enantioselective catalysis and will reappear for several other nanoreactors. The asymmetric aldol reaction between cyclohexanone and *p*-nitrobenzaldehyde is an often-used model reaction, which will be discussed in more detail later. RAFT polymerization led to a series of P(OEGMA-*co*-BTAMA-*co*-*L*-ProMA) copolymers with varying content of the *L*-ProMA catalyst and the structure-directing BTAMA. Most notably, a copolymer with 10 mol% of each (*M*_n_ = 44.5 kg·mol^−1^; *M*_w_/*M*_n_ = 1.58) formed SCPN with *R*_h_ = 8.8 nm in water and reached 99% conversion for the aldol reaction at rt after 24 h with moderate enantiomeric excess (*ee*) of 54%. Kinetic experiments revealed a *k*_app_ of 0.053 s^−1^. Replacing BTAMA with hydrophobic dodecyl methacrylate did not lead to any conversion, suggesting that hydrophobicity was not the driving force for this catalytic reaction. Although the environment of the BTAMA nanopocket controlled the amount of water so that high conversions were reached, the moderate enantioselectivity also indicated that competitive hydrogen-bonding of water molecules interfered during the enamine transition state. A more precise control of the water content is challenging but would be required to reach higher *ee*. Enantioselectivity was improved by controlling the hydrophobicity of the nanopocket. The hydrogen-bond-driven association of BTA allowed for versatile modification of the SCPN core simply by adding functional BTA molecules that would co-associate with BTAMA and together form one nanopocket [59]. As demonstrated on fluorescent 1,8-napthalimide- and *L*-proline-modified BTAs, both could be successfully incorporated into SCPNs at varying contents. Polymer and additive were mixed in THF and dried to a bulk material to ensure homogeneous blending. Redispersion in water gave SCPNs with additive-enriched cores. For catalysis, an average of four *L*-proline-BTA molecules were incorporated into the SCPN and employed in the aldol reaction. The reaction in water at rt for 24 h gave a conversion of 74%, but with a very high *ee* of 95% as compared to the previous system (54% *ee*). Reducing the number of *L*-proline-BTA molecules to two increased the reaction rate, while keeping high enantioselectivity. This demonstrated that the molecular additive was able to arrange within the nanopocket to be shielded effectively from water as compared to covalent attachment to the statistical copolymer (chain segments reduce degrees of freedom). As *L*-proline itself contains hydrophilic moieties, a lower amount of *L*-proline-BTA led to faster and enantioselective reactions due to a better controlled hydrophilicity and, thus, water content within the nanopocket.

SCPN for the reduction of α-diketones to silyl-protected 1,2-diols and the copolymerization of glycidyl phenyl ether with tetrahydrofuran (P(GPE-*co*-THF)) were realized with poly(glycidyl methacrylate-*co*-benzyl methacrylate) (P(GMA-*co*-BzMA)) copolymers [51]. P(GMA-*co*-BzMA) with *M*_n_ < 1 × 10^5^ g·mol^−1^ were prepared by RAFT polymerization and resulted in SCPNs with *R*_h_ = 1.5–2.0 nm in CHCl_3_, while larger SCPNs with *R*_h_ = 17–20 nm were obtained for high *M*_n_ > 1 × 10^6^ g·mol^−1^ prepared by free radical polymerization. The addition of B(C_6_F_5_)_3_ initiated the ROP of the pendent epoxides of PGMA and triggered intramolecular cross-linking to the SCPNs. At the same time, B(C_6_F_5_)_3_ coordinated to the newly formed ether cross-links and served as catalytic site for the bis(hydrosilylation) of benzil with dimethylphenylsilane. The catalytic reaction was done in dichloromethane (DCM) with 0.3 mol% B(C_6_F_5_)_3_ catalyst and gave the product with > 99% conversion in 10 min (TOF = 1980 h^−1^) for the larger SCPNs, which improved further with smaller SCPNs (TOF = 5595 h^−1^). The lower reactivity of larger SCPNs was attributed to the slower diffusion of the substrate through the nanoparticle compartment. The same nanoreactors catalyzed the polymerization of P(GPE-*co*-THF) with *M*_n_ = 55–150 kg·mol^−1^ but broader molecular weight distributions of *M*_w_/*M*_n_ > 2. Controlled polymerization of various methacrylates with narrow molecular weight distributions was demonstrated for SCPNs/FeBr_2_ nanoreactors [60]. A random copolymer with hydrophilic OEGMA (*M*_n_ = 500 g·mol^−1^), hydrophobic dodecyl methacrylate, and urea/aniline moieties for imine cross-linking was synthesized by CRP with RuCp*Cl(PPh_3_)_2_/4-dimethylamino-1-butanol catalyst (Cp* = pentamethylcyclopentadienyl) and ethyl-2-chloro-2-phenylacetate initiator (*M*_n_ = 71 kg·mol^−1^, *M*_w_/*M*_n_ = 1.29). After SCPN formation in water, the core was cross-linked with 2,6-pyridinedicarboxadehyde and the resulting bis(imino)pyridine ligands coordinated with FeBr_2_ in toluene. The halogen catalyst system catalyzed the CRP of MMA, OEGMA (*M*_n_ = 300 and 475 g·mol^−1^), 2-hydroxyethyl methacrylate (HEMA), and methacrylic acid (MAA). All monomers could be polymerized to *M*_n_ < 30 kg·mol^−1^ with *M*_w_/*M*_n_ < 1.30, albeit with comparably long reaction times of 2–4 days. For instance, PMMA_50_ with *M*_n_ < 4.9 kg·mol^−1^ and *M*_w_/*M*_n_ < 1.10 was obtained with 90% conversion after 92 h in toluene at 60 °C. Intriguingly, the core-shielded Fe-catalyst initiated the copolymerization of PMMA with 5–10 mol% MAA in a controlled manner, which is usually very difficult due to chelation and precipitation of transition metal catalysts in presence of carboxylates. Since hydrophilic OEGMA units cover the core, the catalyst was recovered after the reaction by washing the solution with water. Instead of FeBr_2_, Cu-catalyzed ATRP of OEGMA (*M*_n_ = 300 g·mol^−1^), acrylic acid (AA), and *N*-isopropyl acrylamide (NIPAAm) was demonstrated with SCPNs consisting of P(OEGMA-*co*-2-acetoacetoxy ethyl methacrylate) (P(OEGMA-*co*-AEMA)) copolymers [61]. Copolymers with *M*_n_ = 47.1–113.6 kg·mol^−1^, *M*_w_/*M*_n_ = 1.07–1.12, and 12–33 mol% of the Cu-coordinating AEMA were synthesized *via* RAFT polymerization in 1,4-dioxane. SCPN with *R*_h_ < 5 nm were formed in water at a polymer concentration of 1 mg·mL^−1^ and addition of 0.5 equiv. of Cu(II)acetate per AEMA units. UV/Vis confirmed that 0.35 equiv. Cu(II) bound to the *β*-ketoester, forming a core-shell structure *via* intrachain complexation (verified by small angle X-ray scattering). As compared to previous reports (using laccase enzymes), the ATRP of e.g., POEGMA consistently gave narrow *M*_w_/*M*_n_ = 1.1, yet, with relatively low initiator efficiency of *f* < 0.25. A chain transfer agent (CTA) was added to maintain control over the polymerization after initiation with the SCPN. The low *f* and the necessity of a CTA both suggest a reduced accessibility of the active center within the SCPN that would be required throughout the polymerization to activate/deactivate the chain end (absence of CTA led to gel formation). Replacing OEGMA with PMMA, SCPN of P(MMA-*co*-AEMA) complexed with Cu(II) were used for oxidative coupling of terminal acetylenes into 1,3-diynes [62]. The SCPN showed very similar performance to unbound CuCl_2_, e.g., the coupling reaction of propargyl acetate gave the corresponding 1,3-diyne in > 98% yield after 8 h at 60 °C. However, for mixtures of propargyl acetate, 1-octyn-3-ol, and propargyl benzoate, SCPNs selectively catalyzed only propargyl acetate to 98% conversion without any cross-coupling reactions with the other alkynes. The catalytic selectivity could not be reached by unbound CuCl_2_, Cu(OAc)_2_, or Cu(acac)_2_ that instead gave complex mixtures of 1,3-dyines. It was theorized that the nanoenvironment of the SCPN core provided ideal stabilization of the transition state for propargyl acetate coupling over the other substrates reminiscent to the high specificity of enzymes.

The concept of transition metal complexation for polymer folding was also demonstrated on Pd-complex-driven formation of SCPNs employed in Sonogashira coupling reactions [63]. While the repeating unit approach described above utilized statistical intramolecular cross-linking and random positioning of functional groups, the “selective point folding approach” uses mutually orthogonal recognition units at distinct positions along the polymer backbone, presumably leading to better defined SCPNs (Figure 4B). For that, a controlled linear poly(styrene-*co*-4-vinylbenzyl chloride) copolymer (P(S-*co*-VBC) (*M*_n_ = 10.2 kg·mol^−1^, *M*_w_/*M*_n_ = 1.17) was synthesized with NMP. After post-modification of VBC with 4-(diphenylphosphino)benzoic acid, chain folding to SCPN with *R*_h_ = 2.7 nm was induced by intramolecular coordination with dichloro(1,5-cyclooctadiene)palladium (COD was found to be a good leaving group). The Pd-SCPNs successfully catalyzed the Sonogashira coupling of 2-bromopyridine and phenylacetylene albeit with lower conversion and reaction rate as the Pd(PPh_3_)_2_Cl_2_ reference catalyst.

Highly efficient asymmetric sulfoxidation of methylphenyl sulfide with hydrogen peroxide in water was achieved with SCPN containing catalytic titanium(IV) sites [64]. Thermoresponsive random copolymers of NIPAAm and *N*-(1-hydroxy-3-phenylpropan-2-yl)acrylamide (P(NIPAAm-*co*-OxAAm)) (*M*_n_ = 3.1–15.6 kg·mol^−1^, *M*_w_/*M*_n_ = 1.04–1.09) were synthesized *via* RAFT polymerization. The hydrophobic and chiral oxazoline side groups bound Ti(OiPr)_4_ and induced self-folding of the copolymer into SCPNs with *R*_h_ = 2.7 nm by intrachain Ti(IV)-complexation in DCM. Transfer to water compacted the SCPNs further to *R*_h_ = 1.0 nm, while uncoordinated Ti(OiPr)_4_ was hydrolyzed into TiO_2_, which was separated from the SCPNs by filtration. Using 0.1 mol% of Ti(IV)-SCPNs and 1.2-fold excess of H_2_O_2_ to the substrate, methyl phenyl sulfide was converted to (*R*)-methyl phenyl sulfoxide in 97% yield and very high 99% *ee* in water within 60 min (TOF = 990 h^−1^). Similar results were obtained for ethyl, *n*-butyl, and *n*-hexyl phenyl sulfide. Catalytic efficiency was reduced in DCM and with lower oxazoline fraction in the copolymer, both supporting the need of the folded nanopockets for efficient catalysis. The metallo-folded SCPNs could be separated from products by heating the aqueous solution above the cloud point the PNIPAAm, leading to the collapse of the stabilizing corona and precipitation of the SCPNs.

Benzaldehyde condensation to benzoin was reported for SCPN catalysts containing *N*-heterocyclic carbenes (NHC) [65]. The random copolymerization of 4-vinylbenzylethylbenzimidazolium chloride and styrene to P(S-*co*-IL(Cl)) (monomer feed of 5:1, *M*_n_ = 3.1–15.6 kg·mol^−1^, *M*_w_/*M*_n_ = 1.04–1.09) was realized by RAFT. Chain folding was induced by addition of Ag_2_O in DCM that coordinated to two imidazolium units (with an AgCl_2_^−^ counterion). While GPC did not provide conclusive evidence for chain compaction, DLS showed a decrease of *R*_h_ from 5.6 nm to 2.3 nm. The AgCl_2_^−^ counterion was exchanged to a more stable NTf^−^ counterion before the catalyst was activated by thermal decomposition of the poly(*bis*-NHC)-silver complex through refluxing the THF solution at 80 °C for 24 h. This activation was necessary to catalyze benzaldehyde condensation to benzoin as otherwise no conversion was observed. After activation, the conversion increased to 13% for 1 mol% catalyst and further to 32% for 10 mol% catalyst (at higher polymer concentration). Similar catalytic experiments with NHC-complexes in water were also reported for PEO-*b*-PS star-like nanoparticles [66]. The hydrophilic PEO macroCTA initiated the RAFT copolymerization of amphiphilic PEO_114_-*b*-(P(S_24_-*co*-IL(Cl)_11_)) BCP (*M*_n_ = 5.2 kg·mol^−1^, *M*_w_/*M*_n_ = 1.22). Ligation of the Pd(OAc)_2_ to the BCP was done at a polymer concentration of 100 mg·mL^−1^ in DMSO at 120 °C for 24 h. Nanoprecipitation in water gave BCP micelles with *R*_h_ = 3.8 nm (number weighted), where Pd(II) served as supramolecular linker for the core block. The *Suzuki* coupling of iodotoluene and 4-(hydroxymethyl)phenylboronic acid in water at 110°C with 0.1 mol% catalyst proceeded with a 10-times higher turnover number (TON 2900 h^−1^) for the micelles as compared to the molecular catalyst (300 h^−1^), and 29 times higher than the statistical copolymer without a PEO block (100 h^−1^). The high TON only showed minor decline in the second and third catalytic cycle. The scope was expanded employing phenylboronic acids and aryl halides to give seven products with high conversion (>99%).

More recently, SCPNs were reported that efficiently bind CO_2_ and enhance its reactivity for carboxylation of C(sp^3^)-H, C(sp^2^)-H and C(sp)-H bonds [67]. Statistical copolymers of styrene, 4-styryl-di(pentafluorophenyl)borane, and 4-styryl-dimesitylphosphine (feed ratio 0.8:0.1:0.1) were synthesized by RAFT polymerization (*M*_n_ = 60.8 kg·mol^−1^, *M*_w_/*M*_n_ = 1.18). The borane side groups served as frustrated Lewis acceptors and the phosphine groups as Lewis donors (bulky substituents prevented their coupling). Both ligands were required to form dynamic CO_2_-bridged bonds in CHCl_3_, which induced intramolecular chain folding to SCPNs accompanied by a reduction of *R*_h_ from 8.6 nm to 5.7 nm. Coordination by both ligands was confirmed by characteristic shifts of ^11^B- and ^31^P-NMR signals as well as small angle neutron scattering. The activated CO_2_ showed an extraordinary scope for carboxylation as demonstrated on *para*-substituted acetophenones (C(sp^3^)-H) with high yields 91%–98% in 2–6 h, but also *para*-substituted styrene (C(sp^2^)-H) to the corresponding cinnamic acid, and a range of alkynes (C(sp)-H). Since the SCPNs are only folded in the presence of CO_2_ and binding was reversible (after CO_2_ consumption), the SCPNs could be reused for catalysis with further addition of CO_2_ with minor decline of conversion after five cycles. The conversion of CO_2_ into added-value chemicals is an excellent example of how catalytic nanoreactors can aid in cost-efficient production using low energy substrates.

SCPNs were further designed to perform catalytic reactions and tandem reactions together with enzymes inside living cells [68]. A poly(pentafluorophenyl acrylate) was synthesized by RAFT polymerization (*M*_n_ = 23 kg·mol^−1^, *M*_w_/*M*_n_ = 1.05), which is a modular polymer for post modifications [69]. The pentafluorophenyl moiety was replaced by (10-aminodecyl)trimethyl-ammonium chloride and 3-azidopropylamine to give the statistical polyacrylamide copolymer. The azide groups were cross-linked with Ru(bpy)_3_ diyne, resulting in SCPN with *R*_h_ = 3.5 nm. The Ru(bpy)_3_ complex was chosen, because of its photocatalytic ability to reduce azides into amino groups. Azide reduction could be performed in a PBS buffer, but also within HeLa cells (cervical cancer cells) after incubation with RuSCPNs and irradiation at *λ* = 470 nm. The reduction of azides on a dye-precursor produced fluorescent rhodamine110 that could be detected in close proximity to the SCPN (labelled with another dye) as well as in the entire cytosol of the HeLa cells. SCPN catalysts were able to bind the enzyme *β-*galactoidase (*β*Gal) and facilitated their cell internalization so that both catalysts could perform a concurrent tandem reaction. As substrate, a coumarin derivate was synthesized by esterifying coumarin to glucose whose remaining hydroxyl groups were randomly modified with azido phenyl carbonate. The tandem reaction was done in *E. coli* bacterial cells where the Ru(bpy)_3_ catalyst first reduced the azides on the glucose unit, which then could be hydrolyzed by *β*Gal to free the fluorescent coumarin derivate. This work provided a viable example of how a relatively simple copolymer can cooperate with enzymes as artificial organelles for intracellular syntheses.

SCPNs have experienced a sheer boost in the past five years and within this section, we highlighted some notable examples of SCPNs containing transition metal or organocatalysts for catalytic reactions mimicking some aspects of enzymes through synthetic polymer nanopockets. Next to dendrimers, SCPNs are the smallest polymer compartments for catalysis in the range of 1-10 nm. The versatility of monomers in combination with catalysts allows to adjust SCPN properties to suit the environment in which catalysis takes place. The possibility to deliver synthetic catalytic nanoreactors into cells while maintaining their activity inspires to design platform technologies for the on-demand synthesis of complex organic molecules within living cells. The size of SCPNs is certainly advantageous for the reaction rates, while the polymeric character facilitates separation from products through e.g., precipitation. The exact shape and composition of the nanopocket is often unclear, because structure-directing units are randomly located along the polymer chain, which complicates the predictive folding of precise nanopocket architectures. Sequence-defined polymers [70] could be very useful to create more accurate nanopocket environments. Sequence-defined polymers have emerged over the last couple of years, where monomers are positioned along the polymer chain with single monomer precision [71]. Both concepts, sequence-defined polymers and single chain polymer nanoparticles, follow a related aim, i.e., to mimic the ability of proteins to fold into defined 3D nanostructures providing polymer materials with unique physical properties [72]. Introducing catalytic moieties into sequence-defined polymers could bring us even closer to protein and enzyme mimetic catalysts.

## 3. Molecular Polymer Nanoparticles

With the advancement of controlled polymerization techniques, more complex polymer architectures became accessible, including star polymers [22] and cylindrical polymer brushes [23]. These are molecular nanoparticles whose dimension and functionality can be controlled with high precision. They are synthesized either by polymerization of macromonomers (arms first or grafting through), grafting chain end-modified polymers to multifunctional molecules, nanoparticles, or polymer backbones (grafting to), or by grafting polymer arms from multifunctional (or polymeric) initiators (core first or grafting from). Important parameters that define the dimension and functionality of brushes are the arm number *f* controlled by the number of initiating sites and grafting density, as well as the arm length, DP_n_(arm). Initiator-modified surfaces allow the formation of brushes and mixed brushes to create versatile and multifunctional surface coatings [73], e.g., for the immobilization of enzymes as demonstrated on the oxidation of glucose by glucose oxidase (GOx) [74]. Spherical polymer brushes (SPBs) are brush-carrying nanoparticles synthesized by surface-initiated polymerization from preformed latex particles (100 nm core) that have been utilized for catalysis as well [75]. Like for other polymer architectures, the choice of monomer chemistry provides reactive sites for the covalent attachment of one or more catalysts in predetermined locations, but also for sol-gel reactions in compartments to form metal/metal oxide nanoparticles or multimetallic hybrids.

For instance, star polymers with Ru catalysts in the core were synthesized with the arm first method in a one-pot procedure for the oxidation of alcohols [76]. Linear PMMA was first polymerized with metal-catalyzed radical polymerization to a conversion of about 90% in the presence of a non-interfering RuCl_2_(PPh_3_)_3_ catalyst, followed by addition of an ethylene glycol dimethacrylate (EGDMA) cross-linker and DPS as monomeric ligand. The living PMMA chain ends copolymerized both EDGMA and DPS to form the star polymer core (*R*_g_ = 9 nm) with an average arm number of *f* = 22 and arm length of DP_n_(arm) ≈ 100. During cross-linking, the Ru-catalyst migrated into the 2–3 nm core and coordinated to the triphenylphosphine ligands. For the Ru-catalyzed oxidation of 1-phenylethanol to acetophenone in acetone, the molecular RuCl_2_(PPh_3_)_3_ gave almost quantitative conversion after about 2 h, while the star polymer catalyst (31 µmol·g^−1^ Ru/polymer) achieved about 85% yield in the same time. The catalytic activity decreased with higher Ru content (58 and 74 µmol·g^−1^ Ru/polymer), which was ascribed to the confinement of Ru within the nanosized star core (5.4 nm), reducing the number of accessible active sites. The densely cross-linked core network probably also limited the diffusion of the substrate to the active site. With a similar strategy, Pd catalysts were site-isolated in the core of a star polymer with NHC ligands to perform Stille and Heck reactions [77]. NHCs have become prominent ligands in transition metal catalysis as they demonstrate high stability against moisture, temperature, and air. Linear polystyrene (PS) arms were synthesized with NMP and cross-linked to a star polymer with divinylbenzene (DVB) and VBC. The star polymers had a hydrodynamic diameter of *R*_h_ ∼ 6 nm in DMF, a *M*_w_∼3.0 × 10^5^ g·mol^−1^, and an arm number of about *f*∼25−30. Unreacted arms were removed by fractional precipitation in benzene/methanol. Nucleophilic substitution of VBC with NaN_3_ provided azides for the attachment of NHC ligands in a copper-catalyzed click reaction. After core loading with PdCl_2_ and 3-chloropyridine, the star polymers contained 0.15 µmol·g^−1^ Pd/polymer, which efficiently catalyzed the cross coupling of iodobenzene or *p*-nitrobenzene iodide with various olefins in a microwave reactor. For instance, the Stille reaction of iodobenzene with ethyl (*Z*)-3-(tributylstannyl)acrylate) gave the product in 90% conversion after 30 min in DMF at 100 °C. While the unsupported catalyst led to accumulation of Pd NPs during the reaction that reduced the catalytic activity, the star polymer catalyst could be reused five times without noticeable loss of activity and only minor leaking of Pd (<1 ppm). The small size of the star polymers rendered them 4 times more effective as compared to Pd/NHC-modified microparticles.

**Figure 5:**
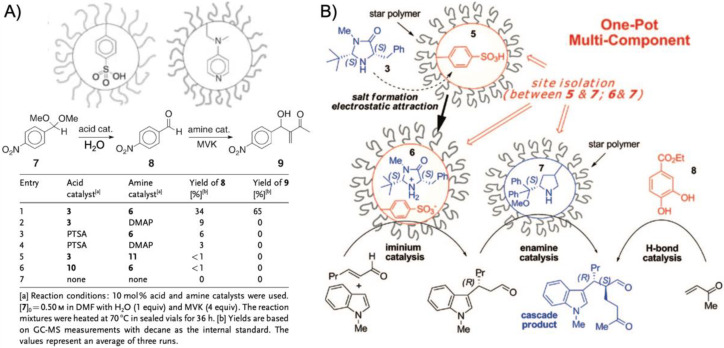
Core-confined acid and base catalysts for cascade reactions. (**A**) PSSA and VEMAP catalysts in star polymer cores performing the acid-base cascade reaction. Reference reactions are given in the table. (**B**) One-pot enantioselective cascade catalysis of site-isolated iminium and enamine catalysts in star polymers. (**A**) adapted with permission of [78]. Copyright 2005 Wiley-VCH. (**B**) reproduced with permission of [79]. Copyright 2008 American Chemical Society.

One benefit of higher arm numbers is that the cores of star polymers do not interpenetrate each other due to steric repulsion of the brush-like shell. It was therefore possible to separately store incompatible catalysts in different cores and mix star polymers without catalyst deactivation. This was demonstrated on two starpolymers containing an acid and a base catalyst in the cores to perform cascade reactions (Figure 5A) [78]. Both star polymers were prepared by NMP of linear PS or PNIPAAm homopolymers followed by copolymerization of styrene and DVB with either phenyl 4-vinylbenzenesulfonate (PVBS) or 4-*N*-(4-vinylbenzyl)oxyethyl-*N*-methylaminopyridine (VEMAP). VEMAP is a derivate of the base catalyst, 4-dimethylaminopyridine (DMAP), whereas thermal deprotection of PVBS to polystyrene sulfonic acid (PSSA) resulted in the acid catalyst resembling *p*-toluenesulfonic acid (PTSA). Mixing both star polymers in DMF allowed the one-pot acid-catalyzed hydrolysis of 4-nitrobenzaldehyde dimethyl acetal followed by the base-catalyzed Baylis–Hillman reaction of 4-nitrobenzaldehyde with methyl vinyl ketone (MVK) with 65% yield of the final product after 36 h (Figure 5A). The same cascade reaction could not be achieved using unsupported DMAP or PTSA or linear copolymers as catalysts. Addition of either DMAP or PTSA to the star polymers quenched the complimentary catalyst, as small molecules were able to diffuse into the cross-linked core network (much like the substrates). This proved the benefit of site isolation of catalysts in star-polymers. In a subsequent work, this acid/base system was developed further by replacing VEMAP with an *L*-proline catalyst for asymmetric acid-base cascade catalysis (Figure 5B) [79]. A MacMillan imidazolidinone organocatalyst was added able to diffuse into the PSSA core to form an iminium ion catalyst bound by electrostatic attraction. The catalyst system enabled the multistep one-pot reaction involving the nucleophilic addition of 2-hexenal to *N*-methyl indole (iminium catalysis) followed by the *L*-proline-catalyzed Michael addition of MVK (enamine catalysis) to the final cascade product with 80% yield and with >99% *ee* in 48 h (89% yield with H-bonding co-catalyst). The reaction proceeded in organic solvents at −30 °C to −40 °C followed by heating to rt. Stereoselectivity for the product could be fully controlled by variation of catalyst chirality. Formation of the cascade product could barely be observed when star polymers were replaced with linear polymer analogues or unsupported catalysts, which again confirmed the protection of catalysts by the star core that prevented their mutual deactivation.

Much like for linear BCPs, controlled polymerization techniques give access to star polymers with AB core-shell and ABC core-shell-corona architecture with precise control over both chemistry and dimension of the core and shell compartments. The spatially defined core of star polymers is attractive to template the growth of inorganic nanoparticles as recently demonstrated on several spherical star BCPs synthesized from multifunctional cyclodextrin initiators [80]. For catalysis, Au NPs were grown in situ in double-hydrophilic unimolecular PAA-*b*-PNIPAAm diblock star nanoreactors [81]. The PAA-*b*-PNIPAAm arms were grafted from halogenated cyclodextrin *via* sequential ATRP of *t*BA and NIPAAm. The P*t*BA core was hydrolyzed to PAA with trifluoroacetic acid. The Au NPs were grown in the core confinement by complexation of auric acid to the carboxylic acid groups of PAA and reduction with *tert*-butylamine borane. The PNIPAAm-capped Au NPs displayed two distinct cloud points above the lower critical solution temperature (LCST), influencing the catalytic activity. The temperature-dependent catalytic activity was shown in the model catalytic reduction of 4-nitrophenol to 4-aminophenol in the presence of NaBH_4_. Below the cloud point (25 °C), the PNIPAAm corona is expanded and the substrate could easily diffuse to the Au NPs surface, which resulted in a *k*_app_ of 4.34 × 10^−3^ s^−1^. However, as the temperature increased above the LCST (50 °C), *k*_app_ decreased to 3.28 × 10^−5^ s^−1^ due to the collapse of the PNIPAAm corona and the hindered diffusion of the substrate to the NP surface. For the latter, PNIPAAm homopolymer was added to the system to facilitate the aggregation of the PNIPAAm-capped Au NPs.

**Figure 6:**
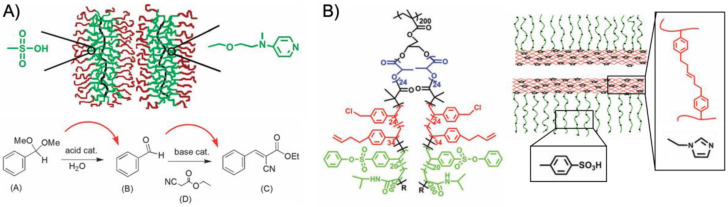
Cascade catalysis with bottlebrushes. (**A**) Acid and base catalysts site-isolated in two bottlebrushes. (**B**) Hollow brushes with base catalyst in the cross-linked shell and acid catalyst on the stabilizing corona. Adapted with permission of [83] and [84]. Copyright 2014 and 2018 Royal Society of Chemistry.

Cylindrical polymer brushes can be seen as the 1D extension of star polymers with essentially the same tailoring capabilities for core and shell [23]. Core-shell brushes have been extensively researched as containers for the growth of inorganic nanocrystals [82]. The utility of core-confined bottlebrush copolymers for catalysis was demonstrated for the site isolation of PSSA and VEMPA catalysts to perform a one-pot acid-base cascade reaction (Figure 6A) [83]. RAFT polymerization of GMA afforded polymer backbones with *M*_w_/*M*_n_ < 1.1 and overall length of 140 units. After hydrolysis of the pendent epoxides and esterification with CTAs (ca. 63% modification), the core-shell brush was grafted from the backbone also by RAFT copolymerization of PVBS (or VEMAP) and 4-(3-butenyl)styrene (BS), and PNIPAAm as a stabilizing outer shell. The BS comonomer is a clever choice as cross-linker, because only the vinyl bond participates in the RAFT polymerization. The 3-butenyl double bond could later be cross-linked in a metathesis reaction. The anisotropic but relatively short bottlebrushes catalyzed the hydrolysis of benzaldehyde dimethyl acetal followed by a Knoevenagel reaction with ethyl 2-cyanoacetate resulting in 87% yield of the cascade product. The reaction was performed in DMSO/H_2_O (40:1) at 70 °C for 48 h (Figure 6A). Control experiments showed that non-cross-linked bottlebrushes or unsupported catalysts led to mutual quenching and no cascade product was formed. To reduce the diffusion path of the reactants between both bottlebrush nanoreactors, a new nanoreactor was designed containing both acid and base catalysts site-isolated within a hollow core-shell-corona brush (Figure 6B) [84]. In this case, ABC triblock terpolymer arms were grafted from the PGMA backbone, starting with lactic acid (LA) *via* AROP to create the sacrificial core. PLA was capped with a CTA to polymerize the VBC/BS shell and the VBS/NIPAAm corona. The final PGMA-*g*-PLA-*g*-P(VBC-*co*-BS)-*g*-P(VBS-*co*-NIPAAm) brush precursor was then transformed into nanotubes by cross-linking the BS units, hydrolysis of PVBS to PSSA, modification of VBC to azide, and click-attachment of 1-(2-(prop-2-yn-1-yloxy)ethyl)-1H-imidazole (PEI) as base catalyst. The organic nanotubes then featured a site-isolated sulfonic acid catalyst in the corona and an imidazole base catalyst in the cross-linked shell. The cascade nanoreactor successfully converted benzaldehyde dimethyl acetal into ethyl *trans*-α-cyano-cinnamate with > 99% yield in water at rt after 24 h.

Molecular polymer nanoparticles are suitable nanopockets for catalytic reactions in the form of star polymers or cylindrical polymer brushes. The enticing feature of such systems is the ability to control shape and size with molecular precision, but also the covalent site isolation of catalysts and functional groups within their interior. The polymeric nanocompartments exhibit intrinsic rigidity while entropic chain repulsion prevents particle interpenetration. Overall, examples for polymer brushes as catalyst supports are still rare, which might be attributed to the multi-step synthesis or challenging synthesis conditions in some cases. Despite being a relatively young field, judging from the increasing number of publications, polymer brushes are on the rise due to their unique properties [23], and AB or ABC bottlebrushes could become valuable scaffolds for advanced multistep cascade reactions.

## 4. Block Copolymer Micelles

Unlike the previously discussed polymer compartments that are covalently bound as one molecular nanoparticle, micelles are supramolecular nanocompartments that spontaneously self-assemble from amphiphiles. Micelles have long been used to accommodate catalysts in the hydrophobic core compartment to transfer and concentrate hydrophobic reactants while shielding the reactive center from the environment. Small molecular weight surfactant micelles have several properties that are very beneficial for catalysis [85]. Surfactants are available in large quantities permitting immediate scale up, the micelle core is typically dynamic (liquid at rt) providing reasonable diffusion constants, and the core size is on the order of few nanometers, increasing the interfacial area with the continuous phase. Micelle catalysis is a highly active field and has recently been used for Pd-catalyzed Sonogashira coupling [86], Rh-catalyzed C-H arylation [87], and for one-pot cascade catalysis of metal-complex chemo- and enzyme bioconversions under aqueous conditions [88], amongst others. Modifications to the chemical structure of small molecular weight surfactant are however limited, as these alter the self-assembly behavior and physicochemical properties. BCP micelles are thus very attractive alternatives, as they are long-term stable and their dimension can be tuned in the range of 10–100 nm simply by increasing the molecular weight of the BCP. The extensive toolbox of polymer chemistry further provides opportunities to equip BCP micelles with a variety of functionalities, e.g., mechanical stability for recycling (selective cross-linking), stimuli responsiveness to external conditions (light, temperature, pH, salinity, or other fields), and catalytic properties by growing inorganic nanoparticles in the core and copolymerization of transition metal or organocatalysts [11,12,13,89]. Further, catalytic activities and conversion rates can be enhanced when the catalyst is immobilized within the nanoconfinement of the hydrophobic core that serves as reservoirs for hydrophobic reactants, increasing the local concentration of catalyst and substrate at the same time.

**Figure 7:**
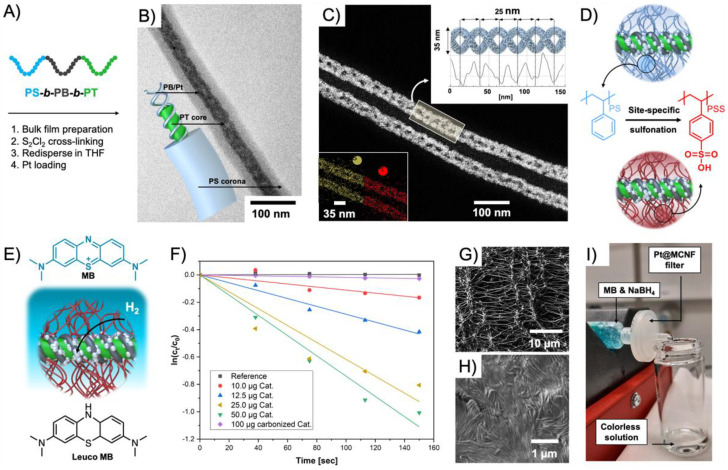
Polystyrene-*block*-polybutadiene-*block*-poly(*tert*-butyl methacrylate) (PS-*b*-PB-*b*-PT) template for organic/inorganic Pt hybrids. (**A**) Nanostructure fabrication process. (**B**) TEM of multicompartment nanofiber with Pt double helix and PS corona. (**C**) STEM of Pt double helix, grey scale analysis of the marked areas, and EDX analysis (yellow: S; red: Pt). (**D**) Scheme of PS sulfonation to PSS. (**E**) Scheme of the catalytic degradation of MB to Leuco MB. (**F**) Ln(*c*_t_/*c*_0_) vs. time without catalyst (black), *c*_cat_ = 10 mg·L^−1^ (red), 12.5 mg·L^−1^ (blue), 25 mg·L^−1^ (yellow), and 50 mg·L^−1^ (green); reference sample Pt/C at 100 mg·L^−1^ (purple). Initial concentrations are: [MB] = 2.0 × 10^−5^ м, [NaBH_4_] = 2.0 × 10^−2^ м. (**G**) SEM image of 0.45 µm PTFE syringe filter and Pt@MCNFs-coated filter surface (**H**). (**I**) Flow set-up to catalyze MB in water using modified syringe filter at a flow rate of 1 mL·h^−1^. Adapted with permission of [93]. Copyright 2020 American Chemical Society.

Although at the heart of this review, we summarize polymer compartments hosting transition metal or organocatalysts, BCP nanostructures are also useful templates for catalytic metal nanoparticles as demonstrated already over two decades ago on the preparation of Pd colloids for the Pd-catalyzed Heck reaction of aryl halides with alkenes [90]. A series of polystyrene-*block*-poly(4-vinylpyridine) (PS-*b*-P4VP) BCPs was synthesized with anionic polymerization (*M*_n_ = 17.5–28 kg·mol^−1^, *M*_w_/*M*_n_ = 1.04–1.15) and assembled to BCP micelles in selective solvents for P4VP (THF, toluene and cyclohexane). P4VP units in the core coordinated a Na_2_PdCl_4_ precursor, which was subsequently reduced to Pd(0) with superhydride (LiB(C_2_H_5_)_3_H). The Pd/micelle system catalyzed C-C-coupling reactions of haloarenes and olefins, where 4-bromoacetophenone and styrene was investigated in more detail. While Pd/micelles led to conversions of > 90% in toluene in 48 h, which is comparable to a Pd(OAc)_2_/tri-*o*-tolylphosphine Heck catalyst, colloidally dispersed Pd/C showed much lower activity. The highest activity was obtained for small micelle cores (27 nm vs. 64 nm) and small Pd NPs. The system showed an increased lifetime and stability as compared to surfactant micelles, and efficiently suppressed Pd NP aggregation to maintain the catalytic activity of the core-confined Pd NPs. Loading of BCP micelles with nanoparticles typically results in the growth of multiple NPs due to a larger number of nucleation sites. In order to grow one NP that completely fills the volume of the core, star polymers were rather successful as discussed in the previous section. To cover the rapidly developing field of polymer/metal (oxide) nanoparticle hybrid structures for catalysis would go beyond the scope of this review and was recently comprehensive summarized in Ref. [91]. We will highlight two examples that make use of ABC triblock terpolymers to form colloidal templates for metal NPs. ABC triblock terpolymers are able to self-assemble into a variety of multicompartment nanostructures with complex geometries of the microdomains. With proper polymer chemistry, they serve as templates for inorganic nanostructures relevant for catalysis and energy conversion [92], as recently also exemplified on hybrid Pt doubles helices using polystyrene-*block*-polybutadiene-*block*-poly(*tert*-butyl methacrylate) (PS-*b*-PB-*b*-PT) templates [93]. The triblock terpolymer was synthesized *via* anionic polymerization with weight fractions that resulted in PT cylinders with PB helix on the surface, and a stabilizing PS corona. H_2_PtCl_6_ was loaded into the helix and reduced with ethylene glycol into hybrid Pt-loaded multicompartment nanofibers (Pt@MCNFs) (Figure 7A,B). The double helix structure was verified by STEM (Figure 7C), electron tomography, AFM, and SEM. Carbonization into Pt NP-decorated nonwoven networks (Pt/C) was followed in situ in TEM by heating to 1000 °C. For catalysis in water, the PS corona was postmodified to PSS in a mild and site-specific sulfonation process (Figure 7D). The water-dispersible Pt@MCNFs catalyzed the reduction of methylene blue (MB) to colorless Leuco MB in presence of NaBH_4_ (Figure 7E). A complete discoloration of the MB solution was reached with 50 mg·L^−1^ catalyst in 3 min corresponding to a *k_app_* = 7.4 × 10^−3^ s^−1^ (green) (Figure 7F). Colloidally dispersed Pt/C showed comparably low *k_app_* = 0.1 × 10^−3^ s^−1^. A 0.45 µm PTFE syringe filter was coated with 25 µg of the water-dispersible Pt@MCNFs to form hierarchal network of Pt@MCNFs on top of the filter network (Figure 7G,H). An MB solution that was passed through the catalytic syringe filter at a flow rate of 1 mL·h^−1^ eluted completely colorless (Figure 7I).

Due to the high specific surface area of NPs, a general problem involved in heterogeneous catalysis with NPs is to efficiently immobilize/stabilize the NPs while maintaining their catalytic activity. Therefore, a “*tea-bag*” like catalyst system was designed, where the Au NPs are stabilized against agglomeration in patches of a micelle corona of ABC triblock terpolymers, while preserving their catalytic activity and recyclability [94,95]. Micelles with crystalline core and patchy corona were prepared from polystyrene-*block*-polyethylene-*block*-poly(*N,N*-dialkylaminoethyl methacrylamide) (SEDxA) triblock terpolymers *via* crystallization-driven self-assembly (CDSA) [96]. The amine-block was obtained through post-polymerization modification of PMMA by amidation with *N,N*-diisopropylethlenediamine (DiPEDA) and *N,N*-dimethylethylenediamine (DMEDA). Coaxial electrospinning of PS homopolymer (*M*_n_ = 1.4 × 10^6^ g·mol^−1^) together with a dispersion of patchy worm-like crystalline-core micelles (wCCMs) or spherical-core crystalline micelles (sCCMs) provided a micelle-modified nonwoven fiber template [97]. These nonwovens were loaded with catalytically active, citrate-stabilized Au NPs by a simple dipping process where Au NPs selectively migrated into the amine corona compartment. The impact of the micelle shape, patch size, and their reuse were tested in 10 catalytic cycles for the alcoholysis of dimethylphenylsilane with *n*-butanol. The reaction proceeded at rt for 7 h and gave the product in quantitative yields. The high reaction rate as compared to reference systems was attributed to the accessibility of separated AuNPs within the patches of the nonwoven surface that prevented diffusion limitation and provided a high surface area for the catalytic reaction at the same time. CDSA was also used to grow micellar poly(ferrocenyldimethylsilane)-*block*-poly(2-vinylpyridine) (PFS-*b*-P2VP) brushes from the surface of silicon wafers [98]. The P2VP corona is known to coordinate Au NPs, which was used for the reduction of 4-nitrophenol to 4-aminophenol with NaBH_4_. The catalytic activity was retained after 15 reaction cycles, while the catalytic substrate could be simply washed and reused.

**Figure 8:**
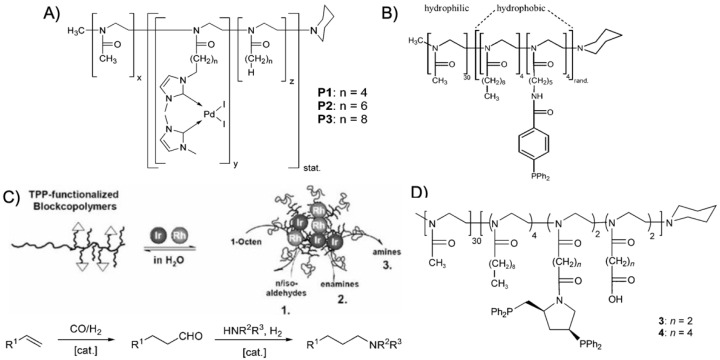
PAOx BCPs for micellar transition metal catalysis. (**A**) Bis(1,3-dimethylimidazoline-2-ylidene)palladium(II) diiodide catalyst for Heck reaction. (**B**) Triphenylphosphane (TPP) ligand for Pd-catalyzed Heck coupling. (**C**) Hydroaminomethylation using the BCP in (**B**) with Ir- or Ir-/Rh-catalysts. (**D**) Chiral (2*S*,4*S*)-4-di-phenylphosphino-2-(diphenylphosphinomethyl)pyrrolidine (PPM) ligand for asymmetric hydrogenation. (**A**) reproduced with permission of [101]. Copyright 2005 American Chemical Society. (**B**,**C**) reproduced with permission of [105]. Copyright 2008 Wiley-VCH. (**D**) reproduced with permission of [106]. Copyright 2003 Wiley-VCH.

Amphiphilic BCP are particularly intriguing as they are able to host catalysts in the hydrophobic pocket for catalysis in water. Over the years, poly(2-alkyl-2-oxazoline) (PAOx) polymers that are synthesized by cationic ring-opening polymerization (CROP) have become very attractive building blocks for efficient catalysis in water [99]. The alkyl moiety of AOx monomers can be tuned to a large extend, whereas the length of the alkyl moiety determines the hydrophilicity of PAOx, which is a convenient handle to control its swelling behavior in water. While excessive amounts of water have a negative effect on several catalytic reactions (e.g., enantioselectivity of *L*-proline), controlled amounts of water are still often needed (e.g., acidic hydrolysis). Being able to control the water content in the core while forming a hydrophobic compartment that is accessible for the substrates is therefore beneficial for many reactions [100]. The 2-alkyl moiety further provides ample opportunities to modify the PAOx monomers with diverse ligands for complexation of transition metal catalysts. This was demonstrated for instance on the Pd-catalyzed Heck reaction of iodobenzene and styrene to *trans*-stilbene [101]. The BCPs were synthesized by sequential CROP initiated with methyl triflate in tetrachloroethane and consisted of a longer hydrophilic poly(2-metyl oxazoline) (PMeOx) block (DP_n_ ~ 30) and a short statistical copolymer of AOx monomers (DP_n_ = 3–5), where the 2-alkyl moieties were butyl, hexyl, and octyl spacers. Of these, 50 mol% carried a pendent bis(1,3-dimethylimidazoline-2-ylidene)palladium(II) diiodide catalyst (Figure 8A). The catalyst was directly copolymerized to improve the degree of core modification (as compared to post ligation); the catalyst content within the copolymer was determined to about 65 mol%. Despite the short hydrophobic block, BCPs self-assembled to spherical micelles in water with a *R*_h_ of 10–30 nm. The catalytic Heck coupling of iodobenzene and styrene was conducted in water at 90 °C with a palladium content of 0.67 mol%. The catalytically active Pd(0) was first formed during an induction period (15 min) by reducing Pd(II) with K_2_CO_3_. After 3 h, the conversion to *trans*-stilbene reached 93% with only minor side products (4% 1,1-diphenylethene and 0.5% cis-stilbene). The butyl spacer provided a TOF = 150 h^−1^ that increased considerably to 570 h^−1^ for the hexyl spacer; the increased distance of catalyst to the polymer backbone improved the accessibility of the catalyst. Micelles could be recycled through diethyl ether extraction, which led to a slight decline in performance with each cycle. Although this was attributed to remaining diethyl ether in the micelles core (hindering swelling with substrates), similar loss in performance was also observed for a variety of other recycled micelle systems discussed in this section. The already excellent TOF was pushed further by increasing the temperature to 110°C where the octyl spacer performed best reaching 2700 h^−1^ [102]. BCP micelles remained thermally stable, the induction period shortened to 3 min, and the accessibility of the most hydrophobic octyl core was enhanced. Other substrates were also efficiently coupled, e.g., the Suzuki coupling of 4-bromobenzophenone, 4-bromobenzaldehyde, and iodobenzene with phenylboronic acid (the latter reached a TOF = 5200 h^−1^). PAOx BCPs with triphenylphosphane (TPP) ligands (Figure 8B) were synthesized with varying substitution pattern (*ortho*, *meta*, *para*) through a polymer-analogous reaction of diphenylphosphane to AOx monomers with pendent iodobenzene moieties [103]. Increasing the TPP density on the chain led to a higher reactivity in the Pd-catalyzed coupling for *para*-substituted ligand as compared to the more sterically hindered *ortho*-substitution. The 1,1-diphenylethylene side product likewise increased for the *ortho*-substituted ligand. The PdCl_2_(PPh_3_)-catalyzed Heck coupling of iodobenzene and various alkenes in water was also realized with thermoresponsive micelles with either non-ionic corona (PEO-*b*-PNIPAAm prepared by ATRP) and anionic corona (PSSNa-*b*-PNIPAAm prepared by RAFT) [104]. The BCP micelles were not modified with ligands, but the PdCl_2_(PPh_3_) catalyst and substrates migrated into the hydrophobic core when the solution was heated above the LCST of PNIPAAm. The anionic PSSNa corona provided slightly higher efficiency for catalytic coupling reactions after 4 h in water at 70 °C (NEt(*i*Pr)_2_ as base) than the non-ionic PEO corona. Although a large variety of substrates was tested, most notable were the coupling of iodobenzene with *n*-butyl acrylate (*n*BA) (99%), *tert*-butyl acrylate (*t*BA) (86%), and styrene (98%), or iodonaphthalene with *n*BA (93%), which demonstrated that the BCP micelles cores supported the catalysis. It should be noted that at temperatures of 70 °C, even without BCPs the reaction of iodobenzene with *n*BA gave *n*-butyl cinnamate with a conversion of 43%. Going below the LCST, the product could be removed from the dissolved BCPs.

POAx BCPs were also redesigned for Rh-catalyzed hydroformylation of 1-octene with CO and H_2_ at 50 bar pressure and 100 °C in a two-phase system (oil/water) [18]. The TPP ligand was introduced by copolymerization of 2-[5-(amino-*tert*-butoxycarbonyl)pentyl]-2-oxazoline and 2-nonyl-2-oxazoline (NoOx) as the hydrophobic block. After removing the protective groups, the primary amine was esterified with 4-diphenylphosphanobenzoic acid through Steglich esterification. The Rh(acac)(CO)_2_ catalyst was efficiently bound by the TPP ligands of the hydrophobic core where also the water-immiscible 1-octene accumulated. The high local concentration of catalyst and substrate led to TOF = 3700 h^−1^ for the conversion of 1-octene to nonanal and isomerized isoaldehyde (*n*/*iso* of 3.0), which is about 3 times higher as compared to unmodified BCP micelles where the Rh-catalyst is stabilized in the water phase by triphenylphosphane trisulfonate (polymer concentration 4 × 10^–4^ mol·L^−1^ in both cases). The biphasic reaction is further beneficial to reach high *n*/*iso* ratios with low ligand/Rh ratios of 5, while under homogeneous conditions (toluene) a ligand/Rh ratio of 500 was necessary. This TPP ligand surpassed its NHC predecessor where either good TOF of 1100 h^−1^ were achieved together with *n*/*iso* = 2.6 or higher TOF of 2300 h^−1^ at lower *n*/*iso* = 1.2 [107]. Using the same TPP-modified PAOx BCP, the hydroformylation of 1-octence was extended to a hydroaminomethylation reaction, which is a multistep one-pot cascade reaction [105]. There, 1-octene was first converted to the aldehyde followed by amination and hydrogenation to form a final amine product. The PAOx BCP was dispersed in water together with Ir- or Ir-/Rh-catalysts to form core-shell micelles with a transition metal loaded core (Figure 8C). Control over catalytic activity could be modulated *via* temperature or the Rh/Ir catalyst ratio.

The enantioselective hydrogenation of (*Z*)-*a*-acetamido cinnamic acid to optically active (*R*)-*N*-acetylphenylalanine required chiral ligands [106]. A MeOx monomer with pendent methyl ester groups was copolymerized with NoOx as the hydrophobic block (*M*_w_/*M*_n_ = 1.17). Hydrolysis of the methyl ester and esterification with the chiral ligand (2*S*,4*S*)-4-di-phenylphosphino-2-(diphenylphosphinomethyl)pyrrolidine (PPM) resulted in the PPM-modified BCPs that coordinated the [Rh(cod)_2_]BF_4_ catalyst in water (Figure 8D). The asymmetric hydrogenation of (*Z*)-*a*-acetamido cinnamic acid with H_2_ in water at 25°C resulted in 48% conversion to (*R*)-*N*-acetylphenylalanine in 24 h, yet, with no detectable enantioselectivity. However, the methyl ester of (*R*)-*N*-acetylphenylalanine was obtained with 95% conversion in 20 min with 85% *ee*. The more hydrophobic methyl ester clearly enriched in the hydrophobic core of the PAOx micelles providing better catalyst performance and higher *ee*. Changing the ligand to 2-isopropoxy-5-hydroxyphenyl methylene allowed the formation of a second generation Hoveyda-Grubbs-type catalyst for the ring-closing metathesis reaction of diethyl-diallyl-malonate in water [108]. The micelles accumulated the hydrophobic diene in the core leading to a conversion of 90% in 1 h at 25 °C corresponding to an excellent TON of 390 h^−1^. The same PAOx BCP proved also useful for the hydrolytic kinetic resolution (HKR) of epoxides in water [109]. To achieve high enantioselectivity, the amount of water participating in the Co(III)(salen)-catalyzed HKR needs to be controlled in a narrow window. Co(III)(salen)-dendrimers demonstrated that the HKR of 2-phenoxymethyloxirane to (*S*)-phenoxymethyloxirane can proceed without loss of enantioselectivity [110]. The chiral (*R*,*R*) Co(III)(salen) complex was implemented into PAOx BCPs by esterification of the (*R*,*R*)-salen ligand to pendent carboxylic acid groups in a similar reaction as described above for PPM, followed by complexation with Co(OAc)_2_ under nitrogen atmosphere for 12 h. Excess carboxylic acid groups ensured quantitative modification, while the remaining acid groups enhanced the hydrophilicity of the core. Micelles with *R*_h_ = 10–12 nm catalyzed the HKR of racemic 2-phenoxymethyloxirane into (*S*)-phenoxymethyloxirane in water at rt with 99% *ee* after 4.5 h and the corresponding diol with 89% *ee* using only 0.02 mol% catalyst. This catalyst system thus displayed a 4–16 times higher reaction rate as compared to previous homogeneous catalysis while the catalyst amount was reduced about 25 times. In addition to enhancing catalytic activity, core confinement reduced the premature hydrolysis of epoxide educts. Similar high enantioselectivity was achieved for the less reactive 2-phenyloxirane and 2-(4-chlorophenyl)oxirane, albeit with longer reaction times. Reduced reaction rates after each cycle were attributed to oxidation of Co(III) to Co(IV) and remaining acidic acid from the catalyst regeneration. Another example involved gold-catalyzed cycloisomerization of allenes to dihydrofurans and dehydrative cyclization of acetylenic diols into furans [111]. PAOx BCPs with varying core hydrophobicity were prepared from 2-heptyl-oxazoline and fluorinated tosylate initiators. A series of comparative experiments revealed that core hydrophobicity, corona size, salt concentration, and the type of gold catalyst all influenced the catalytic performance of the nanoreactor. Best conditions were identified for 2 mol% of AuBr_3_ together with 1 mM of BCP and 5M NaCl resulting in > 80% yield for most heterocyclic products.

**Figure 9:**
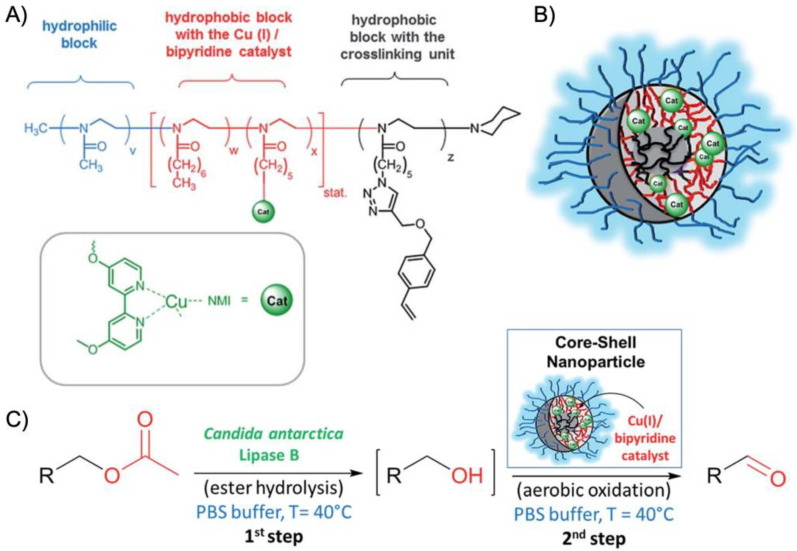
Chemoenzymatic cascade reaction of BCP metal catalyst and enzyme-immobilized polymer beads. (**A**) Chemical structure of the ABC triblock terpolymer. (**B**) Formation of core-cross-linked core-shell NPs with Cu/bpy metal catalyst by microemulsion. (**C**) Two-step cascade reaction of ester cleavage and oxidation for the transformation of esters to aldehydes. Adapted with permission of [118]. Copyright 2017 Royal Society of Chemistry.

More recently, the aerobic oxidation of primary alcohols was realized with bipyridine-functionalized PAOx BCPs [112]. The bipyridine (bpy) ligand was incorporated either as oxazoline comonomer or *via* polymer-analogue reaction to the precursor polymer to create a library of BCPs with different compositions. The catalytic activity of the micelles was studied on a co-catalyst system of Cu(I)Br and 2,2,6,6-tetramethyl-1-piperidine-*N*-oxyl (TEMPO). The micellar (bpy)Cu(I)/nitroxyl co-catalyst system with varying compositions and alkyl spacers performed the aerobic oxidation of primary alcohols, e.g., benzyl alcohol to benzaldehyde, in water with conversions > 99% in 3 h under air at rt (1-methylimidazole (NMI) was added as base). Unlike previous examples, one remarkable feature of this polymer support system was that extraction enabled separation of products and catalysts with no significant decrease in catalytic reactivity even after five cycles. The core-shell structure of BCP micelles also allows the separate storage of two catalysts, e.g., in core and corona (or core and continuous phase). The oxidation of benzyl alcohol to benzaldehyde in water was demonstrated on a library of amphiphilic star BCPs [113]. They were synthesized with a NMP initiator-modified tris(benzyltriazolylmethyl) amine (TBTA), off which first pentafluorostyrene (PFS), 2,2,3,3,4,4,4-heptafluorobutyl acrylate (PFBA) or 1*H*,1*H*,2*H*,2*H*-perfluorodecyl acrylate (PFDA) was polymerized to form a hydrophobic core followed by a hydrophilic *p*-oligo(ethylene glycol) styrene (OEGSt) or OEGMA shell. Molecular weights were kept below *M*_n_ < 15 kg·mol^−1^ with *M*_w_/*M*_n_ < 1.3. According to DLS, the *R*_h_ were larger than 10 nm, reaching even 60 nm in some cases, suggesting that the star-BCPs did not form molecular polymer nanoparticles as described in Section 3, but they self-assembled into micelles and vesicles as verified by AFM and TEM. The CuSO_4_/TEMPO systems catalyzed the oxidation of benzyl alcohol to benzaldehyde in combination with DMAP as co-catalyst in water at rt. From a variety of systems, the highest conversion of 90% was obtained with 1.56 wt.% of the (PFBA_14_-*b*-OEGMA_30_)_3_ scaffold, 2 mol% CuSO_4_, 2.5 mol% TEMPO, and a rather excessive amount of 50 mol% DMAP (all relative to benzyl alcohol). Nevertheless, removing the nanoreactor led to almost no conversion under the same reaction conditions. The same oxidation reaction was investigated for TEMPO-modified micelles synthesized by ROMP-induced self-assembly (ROMPISA) [114,115]. First, a linear copolymer of norbornene derivates with TEMPO and OEGMA was prepared in THF at rt using a Grubs catalyst. Block extension with norbornene in EtOH/THF induced the PISA of micelles with tunable diameters between 10–160 nm (depending on *M*_n_ = 11–250 kg·mol^−1^). The entire synthetic process to catalytic NPs was completed within only few minutes underlining the feasibility for upscaling. For the TEMPO-catalyzed Aneli’s oxidation of alcohols, NaOCl, KBr, and NaHCO_3_ were added to regenerate TEMPO, thereby providing a transition-metal-free catalysis route to alcohol oxidation [116]. The oxidation proceeded with 10 mol% NPs in water (pH 8.6) at 0 °C and reached conversions > 98% after only 1 h. The recyclable nanoparticles displayed similar oxidation rates for isopropanol as previously reported for a molecular TEMPO catalyst in biphasic systems [117]. An interesting alternative application was presented by employing the TEMPO-modified NPs as antioxidants that scavenge reactive oxygen species produced from H_2_O_2_ and HRP, which could be useful in MRI applications. Building on the (bpy)Cu(I)/nitroxyl system discussed for Ref. [112], CalB enzymes were added as incompatible catalysts to perform a chemoenzymatic one-pot cascade reaction in water [118]. Catalytic core cross-linked nanoparticles were prepared from a PAOx-based ABC triblock terpolymer composed of a PMeOx corona, a hydrophobic shell containing the (bpy)Cu(I)/nitroxyl catalyst, and a cross-linkable hydrophobic core (Figure 9A). A microemulsion procedure first created core-shell-corona micelles with *R*_h_~15 nm that were cross-linked with hexanediol dimethacrylate (HDDMA) and AIBN. The (bpy)Cu(I)/nitroxyl carrying nanoparticles (Figure 9B) efficiently catalyzed the oxidation of a range of primary alcohols with >95% conversion and >95% yield. The addition of CalB enzymes immobilized in commercially available polyacrylate beads did not lead to mutual deactivation, and the catalyst mixture was able to perform a multistep one-pot reaction in the PBS buffer solution. Acetylated versions of the primary alcohols were first deacetylated into allylic or benzylic alcohols with >99% conversion by enzymatic ester hydrolysis at 40 °C, followed by oxidation of the alcohol to the corresponding aldehyde by the (bpy)Cu(I)/nitroxyl catalyst with the above-mentioned yields (Figure 9C).

**Figure 10:**
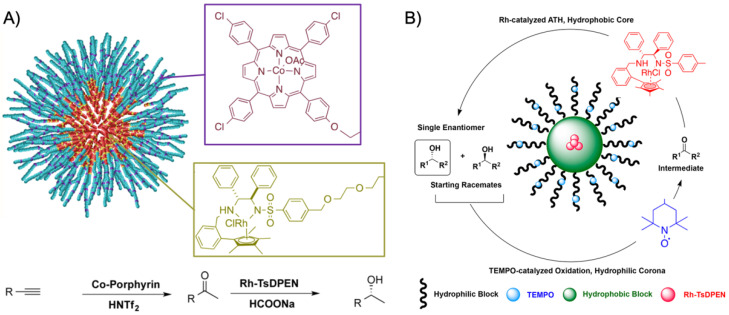
Site isolation of two incompatible catalysts in core and shell of ABC triblock terpolymer core-shell corona micelles. (**A**) Alkyne hydrogenation and asymmetric transfer hydrogenation to chiral alcohols. (**B**) TEMPO-catalyzed oxidation of racemic to ketones and Rh-catalyzed asymmetric transfer hydrogenation. Adapted with permission of [120] and [121]. Copyright 2015 and 2019 American Chemical Society.

A catalytic PAOx-based ABC triblock terpolymer was also developed for the selectively oxidation of alkenes to epoxides [119]. Shell cross-linked micelles (SCM) were produced with a PMeOx corona block, a hydrophobic 2-butyne-2-oxazoline middle block for thiol-yne click cross-linking, and a hydrophobic OAx with pendent methyl ester. The terminal ester was hydrolyzed to carboxylic acids and esterified with hydroxyl-functionalized Ru-porphyrin complexes. Direct dispersion of the BCP in water gave micelles with *R*_h_ = 47 ± 4 nm, which is somewhat large for conventional core-shell corona micelles considering a *M*_n_ = 7.7 kg·mol^−1^. UV irradiation in the presence of 1,5 pentane dithiol and 2,2-dimethy-2-phenylacetophenone cross-linked the micelle shell. Epoxidation of styrene, 2-bromostyrene, 4-vinylbenzylchloride, 1-hexene, and 4-vinylcyclohexane with H_2_O_2_ was conducted with cross-linked and non-cross-linked nanoreactors as well as with unsupported Ru(II)-porphyrin complexes. Both nanoreactors reached a conversion of 94%–99% within 24 h in water at rt with a catalyst loading of 0.35 mol%. Kinetic experiments proved that the final conversion was slightly lower for the cross-linked nanoreactors, which was attributed to the reduced diffusion of entering substrates and exiting products through the cross-linked shell. Recycling and reuse of the catalyst showed no noticeable decline of the performance for the styrene epoxidation. The shell cross-linked micelles could further be equipped with two transition metal complexes site-isolated in shell and corona (Figure 10A) [120]. The PEtOx methyl ester formed the hydrophilic corona after hydrolysis to carboxylic acid, while the hydrophobic NoOx served as inner core. The 2-ethene-2-oxazoline middle block was cross-linked with tetrathiol in a thiol-ene click reaction, leaving some thiol groups for a second click reaction to incorporate the Co(III)−porphyrin catalyst into the shell network. Finally, the corona was esterified with amine-functionalized Rh-TsDPEN. This system was employed in a two-step tandem reaction (Figure 10A). The hydrogenation of phenylacetylene to acetophenone was catalyzed by the 0.13 mol% Co-porphyrin in the cross-linked shell using 2 mol% of the Brønsted acid triflimide as the co-catalyst (in water at 40 °C) followed by the asymmetric transfer hydrogenation (ATH) of acetophenone to (*R*)-1-phenylethanol mediated by 0.07 mol% Rh catalyst. The final product was received in 95% yield with 96% *ee* after 36 h. The combination of Ru-catalyzed ATH and TEMPO-catalyzed oxidation was proven to be useful for the deracemization of initially racemic secondary alcohols (Figure 10B) [121]. Using the concept described above, the Rh-TsDPEN catalyst was clicked into the cross-linked shell of preformed micelles with *R*_h_ = 61 ± 2 nm. Before the incompatible TEMPO catalyst was esterified to pendent carboxylic acid groups in the corona, unreacted thiol groups in the shell were passivated with 1-octene to prevent side reactions during TEMPO modification. While unsupported Rh-TsDPEN catalysts converted acetophenone to (*R*)-1-phenylethanol in water with 99% conversion and high enantioselectivity (98% *ee*) in 12 h, micelles modified with the Rh catalyst reached the same conversion and enantio-selectivity in only 3 h. This remarkable acceleration was attributed to the concentrator effect provided from the nanoenvironment that increased the local concentration of catalysts and substrate. Finally, racemic 1-phenylethanol was converted to (*R*)-1-phenylethanol in the one-pot two-step deracemization by TEMPO-catalyzed oxidation to acetophenone followed by Ru-catalyzed ATH to the corresponding (*R*) enantiomer with 97% conversion in 98% *ee*. Importantly, the reaction time was reduced from 48 h to 12 h with both catalysts located in the same micelle instead of separated in two micelles. This clearly proved that intramicellar diffusion from one catalyst to the next is much faster than intermicellar diffusion due to the shorter distance between catalysts. It is, therefore, generally beneficial to design micellar nanoreactors that are able to accommodate multiple catalysts in the same nanostructure. The deracemization provided high conversion and high *ee* for a range of racemic alcohols with some limitations for solid substrates (58% conversion) and secondary alkyl alcohols (42% *ee*).

Although transition metals are extremely potent catalysts, leaking of partially toxic metals into the reaction solution can be problematic for some products [77]. Over the years, efforts therefore increased to conduct catalytic reactions with organocatalysts. Most prominent are organic acids such as carboxylic, sulfonic or phosphoric acids, as well as organic bases, typically amines. The following examples include one or more organocatalysts into micellar nanoreactors. An early example is given by double hydrophilic PNIPAAm-*b*-poly(*N*-vinylimidazole) (PNIPAM-*b*-PVim) micelles for base-catalyzed hydrolysis [122]. The BCP was synthesized by sequential RAFT/MADIX polymerization and showed thermoresponsive unimer-to-micelle transition at 40°C accompanied by a change from *R*_h_ < 10 nm to *R*_h_ = 34 nm. The PVim block served as base-catalyst for the hydrolysis of 4-nitrophenyl acetate to *p*-nitrophenol. An increased reaction rate was confirmed for micelles as compared to unimers, as the hydrophobic cores supported the solubilization of the substrate. The reaction rate also increased in solvent mixtures that promote micelle formation, confirming an enhanced reaction rate provided by the micelle cores.

The organic base-catalyst DMAP was also included in BCP micelles to create stimuli-responsive hydrophobic pockets for acylation reactions of alcohols in water [123]. VEMAP (compare discussion Figure 5A and Ref. [78]) was copolymerized with styrene by RAFT polymerization into P(S-*co*-VEMAP) with a DP_n_ = 38 and 10 mol% VEMAP. The copolymer was chain extended with PNIPAAm as temperature-responsive corona. BCP micelles with a *R*_h_ = 12.5 nm were formed by dropwise addition of cold water to acetone followed by evaporation of acetone under reduced pressure. During assembly, the temperature was kept below the LCST of 27 °C, as the collapse of the corona would otherwise cause aggregation of the BCP micelles. Covalent binding of DMAP into the hydrophobic domain renders the catalytic site accessible for the substrates while preventing catalyst deactivation through protonation. The DMAP-catalyzed acylation of 1-phenylpropanol with butyric anhydride and co-catalyst *N*,*N*-diisopropylethylamine (DIPEA) gave 95% conversion after 15 min at 5 °C. In comparison, the conversion of 1-phenyl ethanol with acetic anhydride did not surpass 25% under similar conditions. The difference in conversion was attributed to limited mass transport into the micelle core. In case the product is more hydrophobic than the substrates, micelles eventually become saturated. More hydrophobic substrates and reactants are thus required to achieve higher conversions. The thermoresponsive properties of PNIPAAm allowed removing the catalyst by heating the solution above 50 °C and centrifugation of the precipitate. Reuse of the catalyst led to a conversion of 85% after a second cycle, which is an often observed decline in performance for recycled catalyst systems.

**Figure 11:**
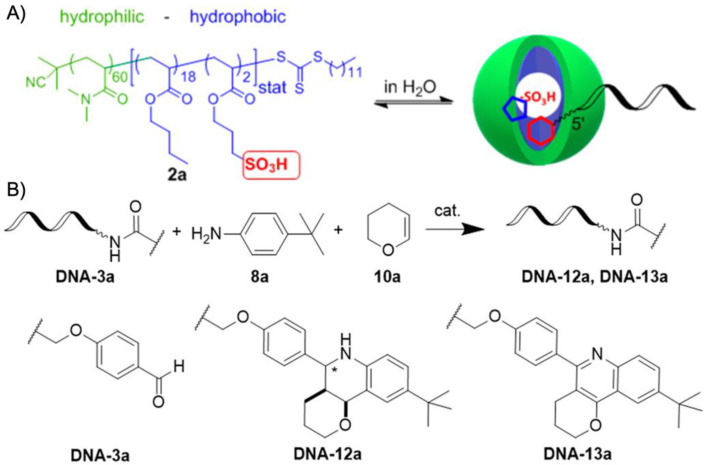
Micellar nanoreactor with Brønsted acid in the hydrophobic domain for the catalysis of DNA-conjugated substrates in water. (**A**) Chemical structure of the amphiphilic BCP and self-assembly to micelles in water with reactive sites for DNA conjugation in the core. (**B**) Povarov reaction of 14mer DNA-benzaldehyde with *p*-*tert-*butyl-aniline and 3,4-dihydro-2H-pyran to a substituted tetrahydrochinoline. Adapted with permission of [124]. Copyright 2019 American Chemical Society.

BCP micelles with strong acid organocatalyst were recently reported to catalyze a large library of DNA-conjugation reactions under mild conditions, i.e., without damaging the DNA itself (e.g., cleavage of purine bases) [124]. The BCP was prepared by sequential RAFT polymerization of hydrophilic *N*,*N*-dimethylacrylamide (DMA) and a statistical copolymer of hydrophobic *n*BA and 3-sulfopropyl acrylate (Figure 11A). The self-assembled spherical micelles in water had a *R*_h_ = 10 nm with the Brønsted acid located in the hydrophobic core. The micellar catalyst was employed in the multicomponent Povarov reaction able to convert a variety of 14mer DNA-aldehydes with *p*-*tert-*butyl-aniline and 3,4-dihydro-2H-pyran to a substituted tetrahydrochinolines in a Diels-Alder reaction (Figure 11B). Quantitative conversion was reached after 6 h at 40 °C, after 4 h at 50 °C, and after 2 h at 60 °C.

All reactants enrich in the micelle core, as confirmed by an increase in *R*_h_ from 10 nm to 50 nm, as well as a morphological transition from spherical to cylinder micelles (confirming considerable increase of the hydrophobic core volume). Without sulfonic acid, the conjugation did not take place, whereas placing it into the micelle corona led to noticeable DNA degradation at elevated temperatures, thereby proving the need to store the strong acid in the micelle core. The library of DNA-conjugates was considerably expanded using a variety of DNA-aldehydes, anilines, and electron-rich olefins. Apart from DNA-aldehyde conjugates, the Brønsted acid within the micelle could perform the Groebke-Blackburn-Bienyamé (GBB) reaction, which created aminoimidazopyridines from aminopyridines and isocyanides. Since the desired DNA-aldehyde may not be readily available, oxidation of the corresponding alcohol is a valuable expansion for the toolbox of DNA-conjugate transformations. Instead of the pendent sulfonic acid group, the same BCP system was extended to carry a (bpy)Cu(I)/TEMPO catalyst in the hydrophobic copolymer as discussed earlier for the alcohol oxidation using PAOx BCPs in Ref. [112]. Using this micellar catalyst, DNA-benzyl alcohol could be oxidized to DNA-benzaldehyde without oxidative damage to the DNA. Both micelle catalysts could even be used in sequence to first oxidize the DNA-conjugated alcohol, followed by the Povarov reaction to the DNA−tetrahydroquinoline conjugate.

**Figure 12:**
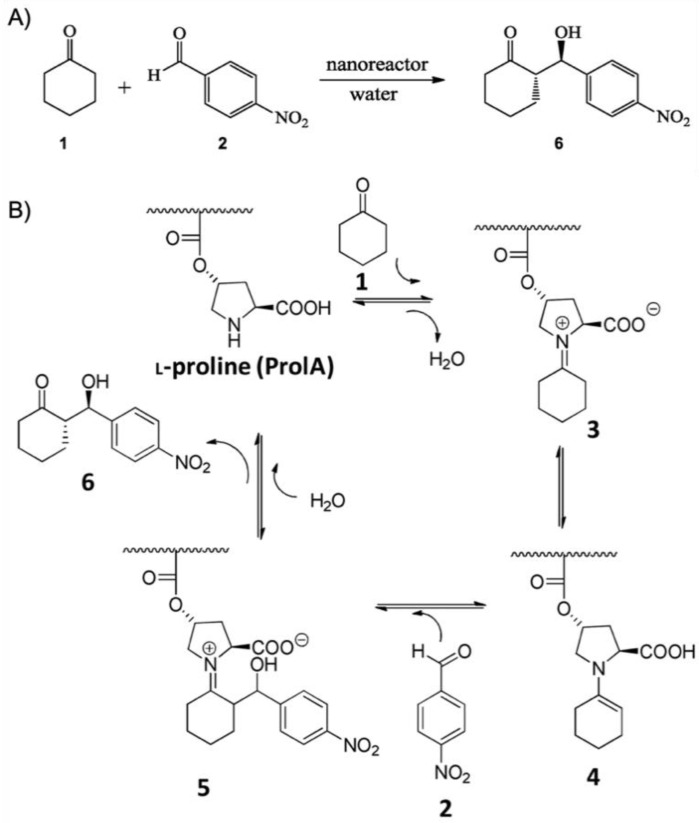
*L*-Proline-catalyzed aldol reaction. (**A**) Aldol reaction of cyclohexanone (1) and 4-nitrobenzaldehayde (2) as discussed for the nanoreactors in the following. (**B**) Individual reaction steps exemplified on a *L*-proline-modified methacrylate. Reproduced with permission of [126]. Copyright 2013 American Chemical Society.

If the organocatalyst inherits a stereocenter, enantioselective catalysis becomes viable also in polymer compartments, as exemplified on *L*-proline-catalyzed aldol reactions [125]. A frequently reported model reaction is the *L*-proline-catalyzed aldol reaction of cyclohexanone and 4-nitrobenzaldehyde to give the stereo- and enantioselective aldol product (Figure 12A). The mechanism involves the following steps (Figure 12B): the nucleophilic attack of the amine of *L*-proline to cyclohexanone (1) and dehydration of the intermediate to the iminium ion (3); deprotonation into the enamine (4) initiates C-C bond formation with *p*-nitrobenzaldehyde (2) to give the iminium-aldol intermediate (5) that is hydrolyzed to the final enantiomeric aldol product (6) while replenishing the *L*-proline catalyst. This reaction will be relevant in the following for several nanoreactors.

**Figure 13:**
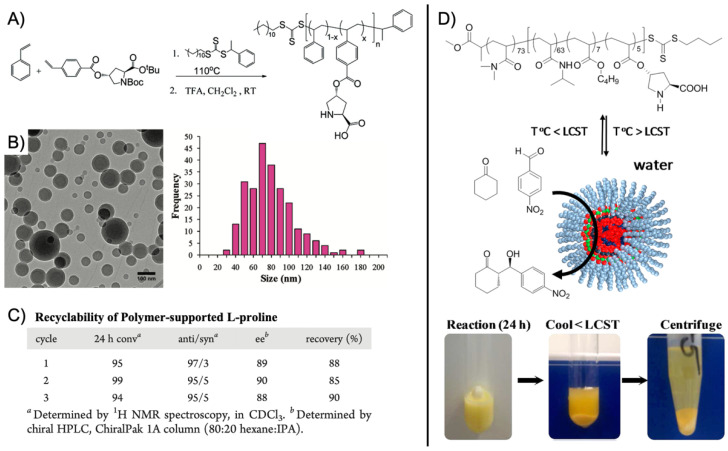
BCP micelles for the asymmetric aldol reaction of cyclohexanone and *p*-nitrobenzaldehyde in water. (**A**) RAFT polymerization of statistical P(S-*co*-ProlA) copolymers and assembly to large compound micelles in (**B**). (**C**) Catalysis with recycled catalyst system. (**D**) Temperature-controlled formation of micelles in water for aldol reaction of. Separation of the soluble catalytic system and insoluble aldol products in water *via* filtration after reaction completion. Adapted with permission of [127] and [126]. Copyright 2011 and 2013 American Chemical Society.

So far, several strategies have been reported to incorporate derivates of *L*-proline base catalysts into BCPs, often by synthesizing a Boc-protected *L*-proline monomer or by post-modification of preformed polymers with functional *L*-prolines [126,127,128,129,130,131]. For instance, the protected 4-vinylbenzoic acid *tert*-butyl/Boc-*L*-proline ester was synthesized by esterification of 4-vinyl benzoic acid with *tert*-butyl/Boc- 4-hydroxy-*L*-proline [127]. RAFT polymerization with styrene in bulk at 110 °C produced copolymers with narrow *M*_w_/M_n_ < 1.10 and a *L*-proline content of about 5 mol% (reflecting the monomer feed) (Figure 13A). The statistical copolymer formed micelles in DMF with low vol% of water with relatively broad size distribution attributed to a monomer gradient along the chain, where higher *L*-proline contents increased the polarity and were theorized to serve as the stabilizing part (Figure 13B). At high catalyst loadings (10 mol% and 20 mol%), both the micelle catalyst and unsupported *L*-proline efficiently catalyzed the aldol reaction (Figure 12A) reaching conversions of > 90% with > 85% *ee* in DMF after 24 h containing 5 vol% water. At low catalyst loading of 1 mol%, the micelle catalyst reached much higher conversion (57%) as compared to the unsupported *L*-proline (12%). When the reaction was carried out without any addition of water, the micelle catalyst still delivered 49% conversion with 91% *ee* compared to 21% conversion with 83% *ee* of the anti-product. According to DLS, polymers were molecularly dissolved in DMF but chains were contracted with a *R*_h_ of only 1.5 nm reminiscent of SCPNs. Addition of 5 vol% water initiated the aggregation into larger structures with *R*_h_ = 80 nm. Judging from the size in TEM, the larger structures could be either assigned to large compound micelles or possibly polymer-rich droplets stabilized by the polymeric surfactant. The micelle catalyst could be recycled and reused without loss of activity or selectivity (Figure 13C). The copolymer design was not ideal for micelle formation as it did depend on the unknown copolymer composition along the chain. A clearer separation of corona and core was realized by RAFT polymerization of an amphiphilic PDMA-*b*-poly(NIPAAm-*co*-BA-*co*-*O*-acryloyl-*trans*-4-hydroxy-*L*-proline) BCP (Figure 13D) [126].

Below 25 °C, the BCP dissolved but maintained nanopockets of hydrophobic BA, which may explain the 56% conversion with 97% *ee*. The high enantioselectivity suggests a good control over water content in the vicinity of the catalytic center or else competitive hydrogen-bonding during the enamine transition state would have reduced enantioselectivity. While the free *L*-proline in DMF/H_2_O (95/5 *v/v*) was significantly slower with loss in stereo- and enantioselectivity at temperatures of 50 °C, BCP micelles formed above the LCST and gave 95% conversion with 96% *ee*. The thermal transition from micelle to unimer enabled the separation of the catalyst from the aldol products by centrifugation. The catalyst was reused simply by heating the solution above the LCST albeit with noticeable lower conversions after each subsequent catalytic cycle attributed to either hydrolysis of the catalyst from the backbone or changes to the enamine transition state rendering the catalyst less effective.

In a related work, POEGA served as the water-soluble corona while the P(NIPAAm-*co*-ProlA) core did not contain any intrinsically hydrophobic component (like BA in the previous example) [128]. Several polymer systems were compared for the aldol reaction in water (containing 5 vol% cyclohexanone): pure *L*-proline or unbound *L*-proline in presence of the BCP did not give any relevant conversions at rt or 50 °C. Without the PEOGMA corona, the P(NIPAAm-*co*-ProlA) copolymer gave 10% conversion at rt, but 76% at 50 °C, which might be attributed to a higher reaction rate at elevated temperatures; the BCP reached 62% conversion at rt and 89% conversion with 96% *ee* at 50 °C. These results are in line with the previous observations, but the origin of the catalytic activity of single chains remained unresolved. To learn more about the effect of core hydrophobicity on catalytic performance, two micelle cores were synthesized for *L*-proline catalysis, one made of PS and one of more polar PMMA [130]. The two *L*-proline monomers described above (Figure 13A,D), ensured statistical copolymerization with styrene and MMA using RAFT polymerization. After chain extension with P*t*BA and hydrolysis to a hydrophilic PAA corona, BCPs micelles with comparable *D*_core_ of 17 ± 3 nm and 19 ± 4 nm were obtained through dialysis from DMF to water at a BCP concentration of 1.0 mg·mL^−1^. Interestingly, the catalytic activity for the aldol reaction increased for both micelle species when the catalyst concentration was decreased from 10 mol% (TON = 9 h^−1^) to 1 mol% (TON = 96 h^−1^), i.e., the reaction rate is 11 times higher with less BCP micelles present. This was attributed to accumulation of reactants in the fewer micelle cores, which plasticized the cores and facilitated accesses to the active sites. The PMMA cores gave the product with lower enantioselectivity (93% *ee* for PS and 85% *ee* for PMMA) attributed to the higher polarity of PMMA that may allow more water to diffuse into the core interfering with the enamine transition state.

pH-responsive micelle catalysts for the aldol reaction in water were realized by a series PEG-*block*-poly(2-(diethylamino)ethyl methacrylate-*co*-*L*-proline acrylate) (PEG-*b*-P(DEAEMA-*co*-ProlA)) BCPs [131]. Copolymerization of DEAEMA and the *tert*-butyl protected ProlA with a PEG macroCTA gave a range of BCPs with various molecular weights and *M*_w_/*M*_n_ < 1.3 (the *L*-proline content was kept below four units per chain in all cases). BCP micelles were assembled in water by direct dispersion at specific pH values. At acidic conditions (pH = 4), DEAEMA is fully protonated and the BCP dissolved. At basic conditions (pH = 9), deprotonation of DEAEMA increased its hydrophobicity and BCP micelles formed with a *R*_h_ > 100 nm. Considering a *M*_n_ < 25 kg·mol^−1^, aggregates are likely not core-shell micelles but larger agglomerates or large compound micelles. The aldol reaction was performed at pH 4, 7, and 9, with 4-hydroxy-*L*-proline, P(DEAEMA-*co*-ProlA), and BCPs. The polymer systems performed best at pH 7 where the pure *L*-proline did not lead to any useful conversion. The higher performance at pH 7 likely originated from self-assembly of BCPs to stable micelles providing a hydrophobic nanopocket for the catalyst with low water contents. The reduced performance at pH 9 was assigned to the agglomeration and partial precipitation of the polymers likely caused by the comparable short PEG block (DP_n_ = 22). As mentioned in the previous examples, a lower conversion was also observed for this system after each recycling step while enantioselectivity remained unchanged.

**Figure 14:**
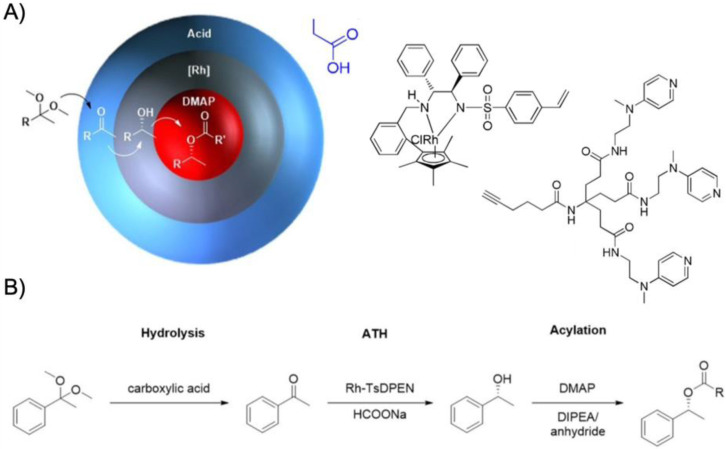
Core-shell-corona micelles with a cross-linked shell inheriting three incompatible catalysts. (**A**) Poly(2-oxazoline)-based core-shell-corona morphology with acid, base, and metal catalyst isolated in corona, core, and shell respectively. (**B**) Three-step reaction for hydrolysis-hydrogenation-acylation cascades of ketals to enantiomeric esters. Adapted and reproduced with permission of [133]. Copyright 2018 Wiley-VCH.

A shell cross-linked nanoreactor inheriting an isolated acid and base catalyst for cascade catalysis was recently reported for ABC triblock copolymers [132]. The PAOx terpolymer was synthesized with CROP of a MeOx monomer with pendent methyl ester that after hydrolysis provides the carboxylic acid in the corona, a 2-butene-2-oxazoline for thiol-ene cross-linking, and a NoOx as the hydrophobic core. The polymerization was terminated with Boc-protected tris(2-aminoethyl) amine catalysts (TREN) to attach the base catalyst at the chain end. After self-assembly to micelles in water, the shell was cross-linked with 1,5-pentanedithiol and a photoinitiator, and the base catalyst was deprotected to create a nanoreactor with base catalyst in the core spatially separated from the acid catalyst in the corona (molar ratio of acid to base = 50:1). The bifunctional nanoreactor was employed in the acidic hydrolysis of benzaldehyde dimethyl acetal followed by the base-catalyzed Henry reaction with nitromethane in a one-pot reaction. While the hydrolysis proceeded with > 99% conversion, the product (mixture of three possible outcomes) was obtained with 86% yield. A series of control experiments employing only the molecular catalysts or unprotected versions of acid and base, all pointed towards a higher performance of the bifunctional catalyst system. As an extension to this work, three incompatible catalysts were incorporated into the core-shell-corona nanoreactor to perform a three-step tandem reaction in one pot [133]. Expanding on the above-described process for core-shell-corona micelles with carboxylic acid in the corona and DMAP base-catalyst in the core, a Rh-TsDPEN complex was additionally immobilized in the shell during the thiol-ene cross-linking step (Figure 14A, compare Ref. [121]). The CROP was terminated with sodium azide for click reaction with an alkyne-modified tri-DMAP later residing in the hydrophobic micelle core. The three-step cascade reaction proceeded in water for 72 h and involved the acidic hydrolysis of benzaldehyde dimethyl acetal to benzaldehyde, the ATH to (*R*)-1-phenylehtanol catalyzed by the Rh-TsDPEN complex, and the DMAP-catalyzed nucleophilic acylation with octanoic anhydride to give the enantioselective product in 60% yield and 98% *ee* (Figure 14B). All three catalysts are spatially isolated from each other, but still in close proximity in their corresponding compartment in a single micellar system, which ensured fast substrate diffusion and conversion to the final product.

**Figure 15:**
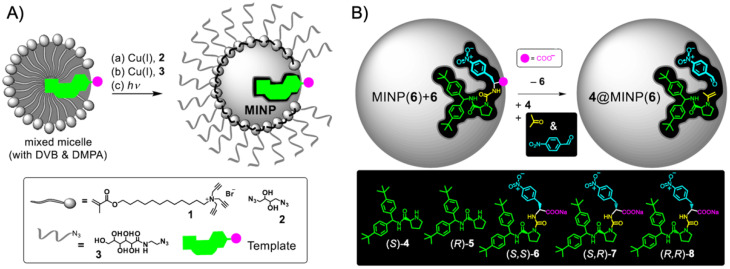
Chiral gating of substrates for asymmetric aldol reactions. (**A**) Templated polymerization of a cross-linked micelle. (**B**) Molecularly imprinted nanoparticles prepared by peptide chemistry. Adapted with permission of [135]. Copyright 2015 American Chemical Society.

A completely different approach to asymmetric catalysis involved the imprinting of cross-linkable micelle cores with chiral pockets providing recognition sites for the substrate in an analogy to the lock and key principle of enzymes. Molecularly imprinted nanoparticles (MINPs) were realized by co-assembly of a tripropargylammonuiumbromide dodecyl methacrylate surfactant together with the substrate as template (Figure 15A) [134]. Free radical polymerization of DVB with the photoinitiator 2,2-dimethoxy-2-phenylacetophenone cross-linked the micelles core, while the propargyl moieties were cross-linked on the surface with diazides, thereby solidifying the entire nanoparticle. For the *L*-proline-catalyzed aldol reaction of acetone with various benzaldehydes, chiral microenvironments were fabricate by peptide chemistry using (*S*)-prolinamide (green in Figure 15B) as the active site and (*S*)-*p*-nitrophenylalanine (cyan in Figure 15B) to facilitate chiral gating of catalyst and substrates (*S*,*S* template) [135]. The *(S)*-prolinamide catalyst and non-imprinted nanoparticles only showed minor activity in water. Addition of increasing amounts of MINPs to a solution containing *(S)*-prolinamide enhanced conversion and enantioselectivity in water peaking at 99% conversion (96% *ee*) for 15 mol% MINPs and 10 mol% *(S)*-prolinamide catalyst. The MINPs were able to safely store the catalyst in the hydrophobic core pockets where it could perform the catalytic reaction. The nanopockets showed high selectively towards the proper catalyst enantiomer with binding constants of 20.4 × 10^4^ M^−1^ for (*S*)-proline and 0.18 × 10^4^ M^−1^ (*R*)-proline. Similarly, the nanopocket was selective to the benzaldehyde derivate as increasingly bulky substituents led to lower conversions, e.g., *p*-chlorobenzaldehyde with similar size gave 95% (98% *ee*), whereas *o*-nitrobenzaldehyde yielded 28% (65% *ee*), and naphthaldehyde only 11% (0% *ee*). By changing the template to naphthaldehyde instead of *p*-nitrobenzaldehyde, naphthaldehyde was catalyzed with 87% conversion (97% *ee*), underlining the potential of the template to accommodate the proper substrate.

**Figure 16:**
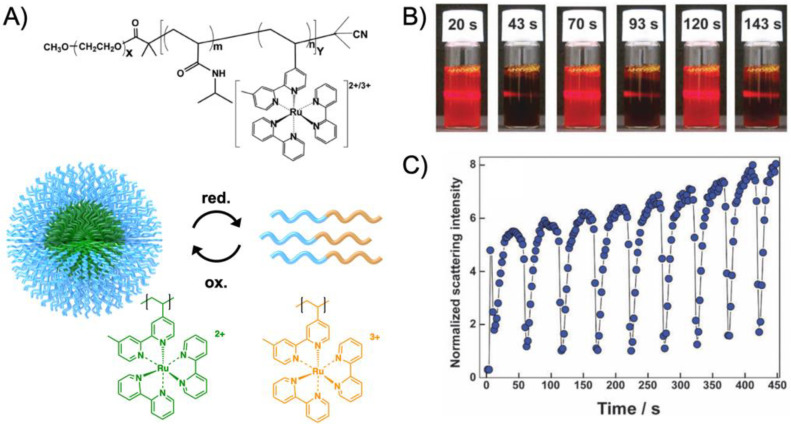
Self-oscillating micelles. (**A**) PEO-*b*-P(NIPAAm-*r*-Ru(bpy)_3_) BCP and redox-responsive assembly/disassembly depending on the oxidative state of Ru(bpy)_3_. (**B**) Time-dependent scattering of the BCP solution synchronized to a BZ reaction. (**C**) Recorded scattering intensity over time showing oscillating formation/dissipation of BCP micelles. Adapted with permission of [136]. Copyright 2013 Royal Society of Chemistry.

Aside from the synthesis of organic molecules or polymers, catalysts in polymer compartments also provide the opportunity to equip nanostructures with advanced functionality, e.g., controlling the shape and form of nanostructures over time through reaction-driven transformations. Self-oscillating micelles based on amphiphilic PEO-*b*-P(NIPAAm-*r*-Ru(bpy)_3_) BCPs were synthesized by RAFT polymerization (Figure 16A) [136]. The BCP was completely water-soluble in the oxidized form (Ru(bpy)_3_^3+^, green), but aggregated in the reduced form (Ru(bpy)_3_^2+^, orange) due to decreased solubility of the core block leading to chain association and micelle formation. Through oxidation/reduction of Ru(bpy)_3_, micelle formation was reversible at 25 °C (LCST of PNIPAAm). The redox-responsive assembly/disassembly ability was coupled to the well-known oscillating Belousov-Zhabotinsky (BZ) reaction to create a self-regulating unimer-to-micelle transition with spatiotemporal control. After addition of malonic acid, NaBrO_3_, and HNO_3_ to an aqueous solution of oxidized BCP (0.5 wt.%), the Ru(bpy)_3_^3+^ complex catalyzed the BZ reaction and was in turn reduced to Ru(bpy)_3_^2+^ triggering the self-assembly into micelles (Figure 16B). The oscillating nature of the BZ reaction led to periodic oxidation/reduction of Ru(bpy)_3_^2+^/Ru(bpy)_3_^3+^, which disassembled the micelles into individual chains and back to micelles. Formation/dissipation cycles were accompanied by a change in scattering intensity, which provided a convenient readout for the completion of each catalytic cycle with a periodicity of approx. 52 s (Figure 16C). By increasing the length of the associating block (*f*_EO_ = 13 wt.%), a very similar transition between BCP chains and polymer vesicles was accomplished [137]. Due to the larger equilibrium radius of the vesicles in the range of micrometers, the structure could be imaged with fluorescence microscopy, while their formation and dissipation could be followed in situ with optical microscopy. After 80 s, capsules had formed through fusion of individual capsules as visible with progressing time. This example demonstrated how catalytic reactions are able to induce changes to BCP morphologies in solution by catalytic reaction-driven formation/dissipation. We will revisit this concept of catalysis-driven spatiotemporal control for polymersomes in the following section.

Overall, there is quite a number of intriguing possibilities to catalyze substrates in either the micelle core, shell, corona, or all of these depending on the BCP micelle design (block composition, block ratios, and catalyst placement). Some advantages are that self-assembly of micelles is relatively straightforward, they are still small with sizes of 10–100 nm, they are structurally stable, and polymeric building blocks can inherit versatile responsive properties. Since BCP micelle are held together with supramolecular forces, swelling agents (such as substrates or products) may plasticize the core and increase chain mobility to a point where micelles melt, merge, and transform. These complications currently limit the scope of BCP micelles as sophisticated multi-catalyst system, because even if catalysts are clearly separated in the beginning (e.g., core and corona), they will be able to react with each other once the micelle is softened. Cross-linking of individual parts of the micelle is one option to counteract this problem. However, cross-linking density affects swelling and may hinder diffusion of the substrate to the catalytic center. If the product itself is more solvophobic than the substrate, it may enrich in the core and prevent substrates diffusion to the catalyst. Although some of these issues have already been approached with innovative nanoreactors as discussed in this section, some challenges still remain that call for more complex micelle systems with multiple compartments each addressing a specific need (see also Section 6).

## 5. Polymer Capsules

While previously discussed polymer supports typically involve the covalent attachment of catalysts to polymers, it is entirely possible to store catalytic compounds within solvent-filled compartments of nanoreactors by co-assembly or encapsulation. Polymer vesicles, also called polymersomes [138], are the most studied solution morphology of amphiphilic BCPs that self-assemble from asymmetric BCPs with large hydrophobic blocks or with ABA block sequence. In an analogy to catalytic processes in organelles, polymersomes can be considered as a multicompartment nanostructure able to separate multiple reactive species from each other in lumen and continuous phase (or the membrane) given that catalysts are large enough to not diffuse through the polymersome membrane. It was recently highlighted that the stiffness of nanoparticles likewise plays an important role for transmembrane diffusion together with particle size and charge [139]. More complex versions of polymer capsules contain responsive channels in the wall that open and close through specific triggers and thereby enhance or suppress access to the catalytic site [140]. Polymer capsules have therefore become one of the most versatile types of nanoreactors as recently summarized in a comprehensive review on polymeric nanoreactors in general [14], and with focus on catalytic reactions in particular [141].

That catalytic reactions can be performed in the confinement of the hydrophobic membrane of polymersomes was shown on the enantioselective cyclopropanation of styrene derivates with ethyl diazoacetate in water [142]. PEG-*block*-poly(styrene-*co*-4-vinylbenzyl azide) (PEG-*b*-P(S-*co*-4-VBA)) with 10 mol% azides and *f*_EO_ = 10 wt.% was synthesized by RAFT polymerization and assembled into polymersomes by slow addition of water to a polymer/THF solution. The polymersome wall was subsequently cross-linked with a C_2_-symmetric alkyne-modified Cu(II)-bis(4-phenyl-2-oxazoline) catalyst in a CuAAC click reaction. The molecular catalyst gave the product in 40% yield with 86% *ee* after 2 h in apolar DCM. While the same reaction in water resulted in only 9% conversion with 30% *ee*, storing the catalytic center in the polymersome wall gave the product in 39% yield with 84% *ee* after only 10 min. Electron withdrawing groups in *para* position of styrene enhanced the yield to 93% (typical for this catalytic reaction), but polar derivates (-NH_2_, -COOH) could not be catalyzed. It was hypothesized that the hydrophobicity of the substrate determines its affinity to the membrane, which is required to enrich reactants in the vicinity of the catalytic center (similar to micelle cores). The larger polymersomes (as compared to micelles) could be easily removed from the reaction solution by filtration.

A more prominent route for catalysis is the encapsulation of reactive species (e.g., enzymes or nanoparticles) that are shielded from negative effects of the environment so that they are able to perform their function within the volume of the polymersome lumen. Polymersomes of poly(*N*-vinylpyrrolidone)-*block*-poly(dimethylsiloxane)-*block*-poly(*N*-vinylpyrrolidone) (PNVP-*b*-PDMS-*b*-PNVP) ABA triblock copolymers were formed by film rehydration [143]. For that, the BCP was dissolved in CHCl_3_ and dried into a thin film onto a glass substrate. During film rehydration with a phosphate saline solution under stirring for 12 h, BCP layers peeled off and rolled up into polymersomes encapsulating the solution and its contents through random entrapment, here laccase (Lac) or HRP. The larger polymersomes (*R*_h_ = 80–110 nm) were separated from the free enzyme with preparative GPC and used as nanoreactors for the enzymatic oxidation of 2,2′-azino-bis(3-ethylbenzthiazoline-6-sulfonic acid) (ABTS) to the radical cation ABTS^+^ accompanied by an increase in absorbance at *λ* = 414 nm. The lower *k*_app_ = 0.18 s^−1^ of the nanoreactor as compared to *k*_app_ = 0.29 s^−1^ of the free enzyme was attributed to the confinement of Lac to the polymersome volume. Importantly, Lac retained 90% of its activity when shielded from proteinase K in the polymersome, while free Lac was deactivated completely. Aside from macromolecular inhibitors, small molecules like sodium azide did not negatively affect Lac activity either demonstrating that the semipermeable membrane of the PNVP-*b*-PDMS-*b*-PNVP polymersomes allowed oxygen to pass through while retaining larger molecules.

**Figure 17:**
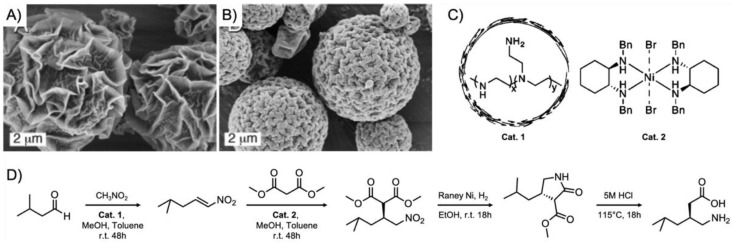
Catalytic reactions in microcapsules. (**A**,**B**) Microcapsules with different wall thickness through interfacial cross-linking of PMPPI and TEPA. (**C**) PEI and nickel-based catalysts. (**D**) Synthesis of Pregabalin from 3-methylbutyraldehyde, nitromethane and dimethyl malonate. Adapted with permission of [144] and [146]. Copyright 2006 and 2007 American Chemical Society.

A heterogenized homogeneous catalyst system was reported for the encapsulation of freely diffusing base-catalyst polymers [144]. A monomer analogue to DMAP, 4-*N*-(4-vinylbenzyl)-*N*-methylaminopyridine (VMP), was copolymerized with styrene to P(S-*co*-VMP) by free radical polymerization and enclosed in microcapsules (*µ*cap). For encapsulation, the P(S-*co*-VMP) was dissolved in CHCl_3_ containing poly(methylene[polyphenyl]isocyanate) (PMPPI) and emulsified with an aqueous solution of polyvinyl alcohol (PVA) and tetraethylenepentamine (TEPA). PVA served as stabilizing surfactant for the oil-in water emulsion, while TEPA cross-linked the PMPPI at the droplet interface to form the *µ*cap shell (Figure 17A). After drying and redispersion in THF, the P(S-*co*-VMP) catalyst remained encapsulated and could be used for the base-catalyzed acetylation of secondary alcohols using acetic anhydride. While the substrates were able to diffuse through the *µ*cap wall, P(S-*co*-VMP) could not pass the membrane and was removed together with the *µ*caps from the solution by filtration. Increasing the wall thickness with increasing amount of TEPA/PMPPI (Figure 17B) reduced reaction rates as diffusion of the substrates became progressively hindered. Although the confined polymer showed lower reactions rates as compared to molecular DMAP catalysts (slower diffusion of the polymer), the P(S-*co*-VMP)/*µ*cap system demonstrated higher reaction rates than DMAP-modified microspheres consisting entirely of a dense polymer network. The site isolation of catalyst-bearing polymers in *µ*caps was further advanced for a one-pot imine formation followed by Michael addition (Figure 17C) [145]. For that, the polyethylene imine (PEI) base-catalyst was encapsulated and site-isolated in *µ*caps through interfacial polymerization of 2,4-tolylene diisocyanate in cyclohexane/methanol oil-in-oil emulsions stabilized by Span 85. An incompatible Ni(II)-bis[(*R*,*R*)-*N*,*N*‘-dibenzylcyclohexane-1,2-diamine]Br_2_ catalyst for Michael addition could be added to the *µ*cap dispersion without mutual deactivation with PEI. The two reactants were used for the tandem reaction of benzaldehyde to *trans*-*b*-nitrostyrene with nitromethane *via* an imine intermediate and further to dimethyl 2-(2-nitro-1-phenylethyl)malonate through Michael addition of dimethyl malonate. Due to the absence of nitroalcohol in the first step, it was postulated in a later work that the reaction mechanism proceeds *via* imine intermediates rather than a base-catalyzed Henry reaction [146]. The importance of site isolation was demonstrated in several reference reactions. Employing only the base-catalyst without subsequent purification of the products resulted in the unwanted side product 1,3-dinitro-2-phenyl-propane, because nitromethane is able to react twice with benzaldehyde. Addition of the nickel catalyst to PEI or other base-catalysts without encapsulation led to mutual deactivation and low product yields. Only the combination of encapsulated PEI and the immediate conversion of the intermediate *trans*-*b*-nitrostyrene with the nickel catalyst gave the final product in 80% yield. By using an enantioselective nickel catalyst, the system was able to produce Pregabalin in 74% yield (Figure 17D) [146]. Pregabalin is an important pharmaceutical drug approved for the treatment of neuropathic pain and epilepsy. The one-pot two-step reaction of 3-methylbutyraldehyde with nitromethane followed by dimethyl malonate gave the enantiomeric product (*S*)-dimethyl 2-(3-methyl-1-nitrobutyl) malonate in 94% yield (72% *ee*). The one-pot reaction was particularly important in this case, as it efficiently suppressed the formation of the previously mentioned side product 1,3-dinitro-2-phenylpropane. Subsequent hydrogenation *via* Raney-nickel and acidic hydrolysis followed by decarboxylation gave Pregabalin in 91% *ee* after re-crystallization. This example impressively demonstrated the potential of carefully designed multi-catalyst systems to reduce processing steps for the synthesis of complex, pharmaceutically useful compounds.

**Figure 18:**
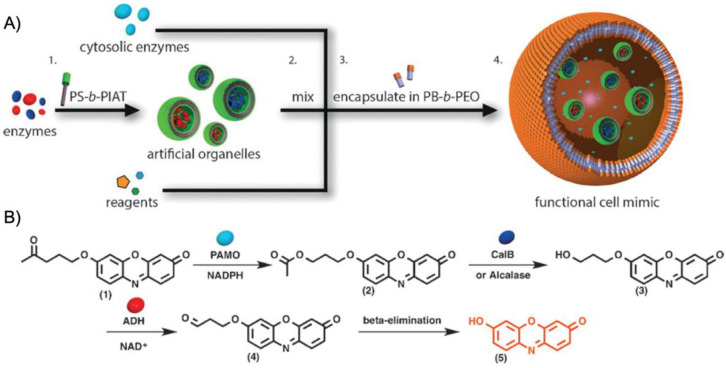
Enzymatic cascade reaction in a polymersome-in-polymersome system. (**A**) Schematic illustration of the encapsulation of the enzymes CalB and ADH in PS-*b*-PIAT polymersomes and subsequently encapsulated by PEO-*b*-PB along with PAMO and substrates in giant polymersomes. (**B**) Four-step cascade reaction of profluorescent 7-((4-oxopentyl)oxy)-3H-phenoxazin-3-one to the fluorescent 7-hydroxy-3H-phenoxazin-3-one. Reproduced with permission of [149]. Copyright 2014 Wiley-VCH.

Instead of confining one catalyst into one container, loading of two polymersomes with different enzymes can be an alternative strategy for one-pot tandem reactions in water [147]. PS-*block*-poly(isocyanoalanine(2-thiophen-3-yl-ethyl)amide) (PS-*b*-PIAT) polymersomes with permeable membranes were prepared by injecting a BCP/THF solution (1 mg·mL^−1^) into water containing either GOx or HRP. After mixing both polymersomes, the addition of glucose and ABTS substrates led to the oxidation of glucose to gluconolactone by the GOx enzyme accompanied by the producing of H_2_O_2_ that, in a second step, was consumed by HRP for the oxidation of ABTS to ABTS^+^. In a preceding work, both enzymes were enclosed in the same PS-*b*-PIAT polymersome, while a third enzyme was located in the continuous phase. HRP resided in the porous membrane, GOx in the lumen and *Candida Antarctica* Lipase B (CalB) was kept outside the polymersome [148]. The substrate 1,2,3,4-tetra-*O*-acetyl-*b*-glucopyranose was deacetylated to glucose by CalB, converted to its lactone by GOx, and the produced H_2_O_2_ aided in the conversion of ABTS to ABTS^+^ by HRP. To go one step further, the porous nature of the PS-*b*-PIAT membrane was also exploited for the separate encapsulation of the CalB and alcohol dehydrogenase (ADH) [149]. The enzyme-loaded PS-*b*-PIAT polymersomes were in turn encapsulated by giant PEO-*b*-PB vesicles together with a third enzyme, phenyl acetone monooxygenase (PAMO) (Figure 18A). These giant polymersome-in-polymersome systems serve as cell mimics capable to exchange reactants between encapsulated polymersomes and to perform a 4-step cascade reaction to produce a fluorescent product (Figure 18B). The profluorescent substrate 7-((4-oxopentyl)oxy)-3*H*-phenoxazin-3-one undergoes a Bayer-Villiger oxidation catalyzed by PAMO with NADPH as cofactor to form an ester, which is subsequently converted by CalB to a primary alcohol. Oxidation of the alcohol catalyzed by ADH takes places in the presence of NAD^+^ to form an aldehyde that is ultimately converted to the fluorescent product, 7-hydroxy-3*H*-phenoxazin-3-one, after spontaneous beta-elimination. This example impressively demonstrated the complexity that can be achieved with comparably simple BCP scaffolds given that both preparation and reactants are carefully chosen.

**Figure 19:**
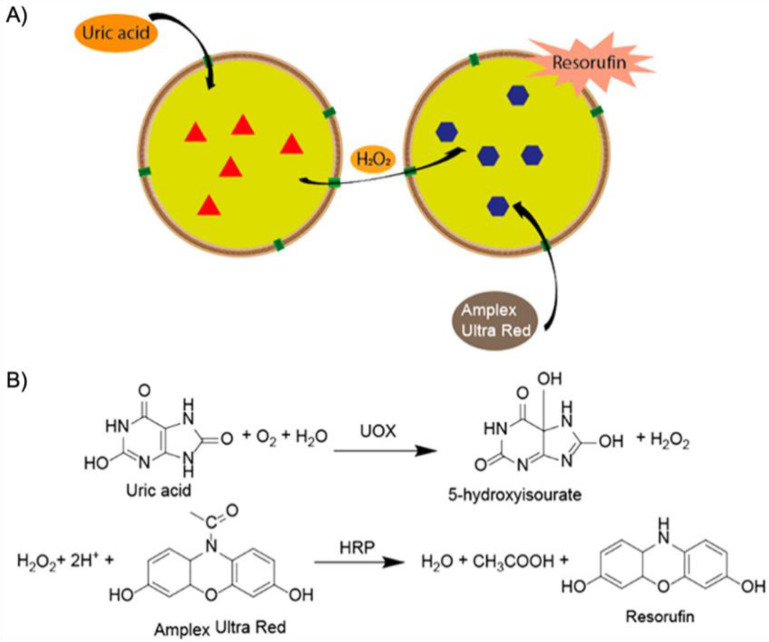
Enzymatic cascade reaction in PMOXA-*b*-PDMS-*b*-PMOXA polymersomes. (**A**) Schematic illustration of the biocatalytic cycle. (**B**) Two-step reaction of uric acid to H_2_O_2_ and subsequently to water catalyzed by UOX and HRP. Reproduced with permission of [150]. Copyright 2014 American Chemical Society.

An enzymatic cascade system for the detoxification of complex media from uric acid was realized by the encapsulation of two different biocatalysts in ABA triblock copolymer polymersomes [150]. Polymersomes of PMeOx-*b*-PDMS-*b*-PMeOx were formed *via* film rehydration in the presence of either fungal uricase (UOX) or HRP (Figure 19A). This non-native combination of enzymes aided in the simultaneous consumption of uric acid and H_2_O_2_, which are associated with pathologic conditions such as gout and oxidative stress. Nanocompartments containing the UOX enzyme converted uric acid to 5-hydroxyisourate and H_2_O_2_ in the first step. In the second step, H_2_O_2_ was catalyzed to acidic water by HRP that converted Amplex red to resorufin (Figure 19B). The cascade reaction with two spatially isolated nanocontainers was evaluated in human blood serum and cells, underlining the bioinspired approach to mimic sequential enzymatic reactions in biological systems.

The conversion of substrates to products is considered to be an energy-dissipative process that can be utilized to induce oscillating volume changes, shape transformation, and directional locomotion. Oscillating self-beating polymersomes were reported for PEO-*block*-poly(NIPAAm-*co*-*N*-aminopropylmethacrylamide) (PEO-*b*-P(NIPAAm-*co*-NAPMAm)) copolymers synthesized by RAFT polymerization [151]. Statistical modification of the primary amines of NAPMAm with *N*-hydroxy-succinimidyl esters of Ru(bpy)_3_ and MMA introduced redox-responsive and cross-linking sites. Polymersomes (*f*_EO_ = 7.1 wt.%) formed by heating an aqueous BCP solution (0.5 wt.%) and 2,2′-azobis(2-methylpropionamidine)dihydrochloride photoinitiator (0.2 wt.%) above the LCST during UV-irradiation to cross-link the membrane. Cross-linking provides mechanical and chemical stability, as otherwise polymersomes would disassemble into single chains upon Ru(bpy)_3_ oxidation [152]. Changing the oxidation state of Ru(bpy)_3_ with an oscillating BZ reaction altered the hydrophobicity of the membrane, which expanded/contracted through de-/swelling mimicking a beating motion. Heterogeneities in membrane cross-linking density caused shape deformation (buckling instability) with pronounced indentations. These indentations relaxed back to spherical polymersomes synchronized with the BZ oscillation.

More drastic shape transformations were observed for polymersomes of alternating copolymers while being driven out-of-equilibrium through the BZ reaction [153]. Poly(2,3-dihydroxybutylene-*alt*-bipyridyl-dithioether) (P(DHB-*alt*-BpyDT) copolymers were synthesized from 5,5′-bis(mercaptomethyl)-2,2′-bipyridine and 1,3-butadienediepoxide *via* step-growth polymerization in the presence of triethylamine in DMF (epoxy/thiol “click” polymerization). The step-growth polymerization naturally delivers *M*_w_/*M*_n_ ~ 2. The redox-responsive Ru(bpy)_3_ was introduced by coordinating *cis*-dichlorobis(bipyridine)ruthenium(II) to the P(DHB-*alt*-BpyDT) in a polymer-analogue reaction. Dropwise addition of water to the P(DHB-*alt*-BpyDT)/DMSO solution (0.1 mL·h^−1^) formed polymersomes that underwent twisting, elongation, and division processes when subjected to the BZ reaction. Unlike the previous example, such disruptive shape transformations were facilitated by the non-cross-linked membrane with low viscosity (hydrated DHB segments) and the overall low *M*_n_ = 5.5 kg·mol^−1^. Although the shapes were not controlled, temporal evolution of solution morphologies provides one of the basic components for life-like synthetic materials.

Recently, polymersomes were demonstrated with self-regulating catalytic performance controlled by enzymatic oscillating reactions [154]. Polymersomes were formed from PEO-*block*-poly[2-(diethylamino)ethyl methacrylate-*co*-2-hydroxy-4-(methacryloyloxy)benzophenone] (P(DMAEMA-*co*-BMA)) [155]. During self-assembly in water through nanoprecipitation, HRP and urease were co-encapsulated into the polymersome lumen. The BMA units were photo-cross-linked to afford a stable, pH-responsive membrane that controled permeation of substrates into the polymersome lumen by swelling (*R*_h_ = 182 nm at low pH) and contraction (*R*_h_ = 101 nm at high pH). Addition of HCl and protonation of PMAEMA allowed ATBS to diffuse into the polymersome where it was oxidized by HRP. Urea on the other hand served as antagonist that gradually suppressed the catalytic oxidation due a steady increase of pH after conversion of urea to ammonia by urease. The catalytic activity of the encapsulated HRP could thus be switched “ON” and “OFF” as well as controlled over time by the amount of added urea. An “ON” and “OFF” state for reaction-driven autonomous movement was also demonstrated for stomatocytes [156], i.e., polymersomes with an accessible cavity. Opening and closing was programmed by addition and consumption of adenine triphosphate (ATP), which controlled the accessibility and activity of encapsulated catalysts [157]. The stomatocytes were formed by co-assembly of PEO-*b*-PS and an azide carrying N_3_-PEO-*b*-PS BCP both synthesized by ATRP. The stomatocyte surface was decorated with polylysine (PLL) in a click reaction (PLL was polymerized from LysZ NCA with dibenzocyclooctyne-amine). The PLL surface brush became more hydrophobic upon electrostatic binding with ATP thereby closing the stomatocyte cavity. In presence of potato apyrase, ATP was catalyzed to adenine monophosphate (AMP) with less binding sites, which gradually opened up the cavity. Loading HRP or Pt NPs [158] into the cavity turned the production of H_2_O_2_ ON and OFF by addition of ATP. Typically, the amount of added H_2_O_2_ and, thus, the amount of released dioxygen propellent, changes the motion from diffusive Brownian motion (closed) to directional motion [159]. However, in the case of ATP sensitive stomatocytes, the ATP concentration and its conversion to AMP served as a time-programmed valve controlling the accessibility of the Pt catalyst and the lifetime of movement speed. Using a similar synthetic strategy, the cup shape of the stomatocytes also allowed selective modification of the inside and outside of the wall [160] essentially resulting in Janus nanocups [161]. For that, N_3_-PEO-*b*-PLA and PEO-*b*-PLA were synthesized by AROP and co-assembled into stomatocytes with azide functionality on the surface. The exterior azides were selectively reduced by tris(2-carboxyethyl)phosphine beads that are too large to enter the cavity, and then tagged with Alexa Fluor 647-NHS dye. The interior on the other hand was exclusively modified with DBCO-carrying catalase and GOx that covalently bound to the remaining azides within the cavity. Catalysis of glucose caused propulsion of the stomatocytes with ballistic motion profiles attributed to oxygen bubble formation that is facilitated by the nanoconfinement of the catalysts.

The difference in number of binding sites of ATP/AMP was also used for the formation of dissipative surfactant vesicles that formed entirely through a reaction-driven process [19]. ATP was shown to bind three C_16_TACN-Zn^2+^ surfactants, which decreases the critical micelle concentration of the surfactant and promotes the formation of vesicles with low interfacial curvature through lowered electrostatic repulsion of the surfactant head groups. If potato apyrase is present in the system, this enzyme simultaneously converted ATP to AMP, which merely binds one C_16_TACN-Zn^2+^ surfactant. The competition between ATP and AMP binding ultimately resulted in decay of the surfactant vesicle once the AMP concentration dominated (conversion of ATP). This example is particularly intriguing as the spatiotemporal controlled state of the vesicle was used to perform a reaction in the hydrophobic domain of the surfactant bilayer as long as the vesicle structure was intact. In this case, 4-chloro-7-nitrobenzofurazan and 1-octanethiol were reacted to 4-nitro-7-octylthiobenzofurazan in a nucleophilic aromatic substitution. Although no catalyst was present in this particular case, the spatial confinement of the hydrophobic bilayer membrane (<10 nm) promoted the reaction giving about 23% yield as compared to 9% yield in the water phase. The vesicle membrane therefore served more as a nanoreactor rather than a catalyst, enhancing the reaction rate through substrate concentration in the nanoconfined environment.

Polymer capsules and polymersomes are prime examples for nanoreactors, as the spatial separation of reactants through a membrane mimics the mode of operation that is typical for organelles. These polymer compartments are able to hold catalytic compounds in their hydrophobic membrane and the solvent-filled lumen, while protecting catalysts from the environment. However, the intrinsic permeability of polymer membranes for small molecules cannot always be suppressed (e.g., plasticization by substrates, osmotic pressure), which imposes limitations on the type of encapsulated catalyst. Colloidal catalysts (enzymes, nanoparticles) or catalyst-modified polymers with larger hydrodynamic radii and reduced diffusivity are thus the most frequently reported examples in catalytic applications. An advanced feature of this polymer support is the great versatility with which permeate selectivity can be enhanced, e.g., by increasing membrane thickness, tuning the cross-linking density in the membrane, or by incorporation of addressable responsive channels that give precise control over transport of substrates into the capsule interior.

## 6. Outlook and Future Perspectives

Besides rapid progress in catalyst development, there are likewise intriguing advancements in soft matter materials that are suitable support materials while adding polymer-specific functionalities [15]. We here give some perspectives that we believe will be beneficial to progress the field of catalysis in polymer supports.

*Inverse BCP morphologies:* This review covered some selected examples of catalysis in self-assembled solution morphologies of BCPs. More recently, the diversity of BCP micelles has been expanded to inverse morphologies, i.e., micron-sized BCP particles that consist of an internal periodic lattice of solvent-filled channels with very large surface area to volume ratios (as compared to polymersomes for instance) [162]. These particles are typically obtained for highly asymmetric BCPs (or lipids) where the hydrophobic part requires such a large volume that it induces a switch in interfacial curvature from quasi-planar (vesicle) to inverted spheres or cylinders termed cubosomes and hexosomes depending on the lattice symmetry. The channel diameter can be tuned through block length in a wide range and is accessible for small molecules, but also nanoparticles and enzymes. Equipping the surface of the channels with catalysts should be interesting to create potent nanoreactors with high interfacial area. This was demonstrated for a *L*-proline-modified lipid cubosome [163]. Boc-*L*-proline was reacted to oleyl amine by EDC/DMAP coupling to give the unsaturated catalytic lipid. After deprotection, the *L*-proline lipid was mixed with monoolein and self-assembled in water with the aid of pluronic surfactants. The *L*-proline-catalyzed asymmetric aldol reaction of cyclohexanone and benzaldehydes reached 99% (80% *ee*) after 4 h in 25 mM HEPES buffer, which was superior to reference reactions involving soluble *L*-proline catalyst that only reached 65% after 48 h. The fast reaction suggested that *L*-proline was facing the solvent-filled channels with easy access of the substrates. The catalytic performance was influenced by a range of parameters, e.g., channel diameter, lattice symmetry, and catalyst content. In another work, the *L*-proline-functionalized lipopeptide, PRW-NH-C_16_, was self-assembled to cubosomes and tested in the aldol reaction of cyclohexanone with *p*-nitrobenzaldehyde giving > 99% yield after 48 h with 89% *ee* [164]. These examples demonstrated the potential to conduct catalytic reactions in lipid mesophases, but they also highlighted the need for the careful design of catalyst-modified lipids to prevent morphological transition; slight changes to the chemical structure of the lipid has drastic consequences for the self-assembly behavior. Unlike lipid mesophases, BCP mesophases should be more robust to modifications of the building block and BCP cubosomes could therefore become versatile scaffolds for efficient catalysis in water. BCP cubosomes already served as templates to create inorganic replica of the channel system with control over pore size from 20–200 nm [165]. An inorganic precursor salt was infiltrated into the channel system and reduced to form a single network replica. Exemplified on ordered mesoporous metal-phenolic networks, PEO_45_-*b*-PS_217_ was self-assembled to BCP cubosomes in water followed by chelation of phenolic epigallocatechin gallate and Fe^3+^ [166]. After removal of the organic template, a self-supporting network remained with pores that could be loaded with enzymes in high weight fractions, e.g., HRP with 486 mg/g and GOx with 362 mg/g. The HRP-loaded bioreactors performed the enzymatic oxidation of amplex red to resorufin in the presence of H_2_O_2_ with better performance as compared to mesoporous silica or the BCP cubosome template.

**Figure 20:**
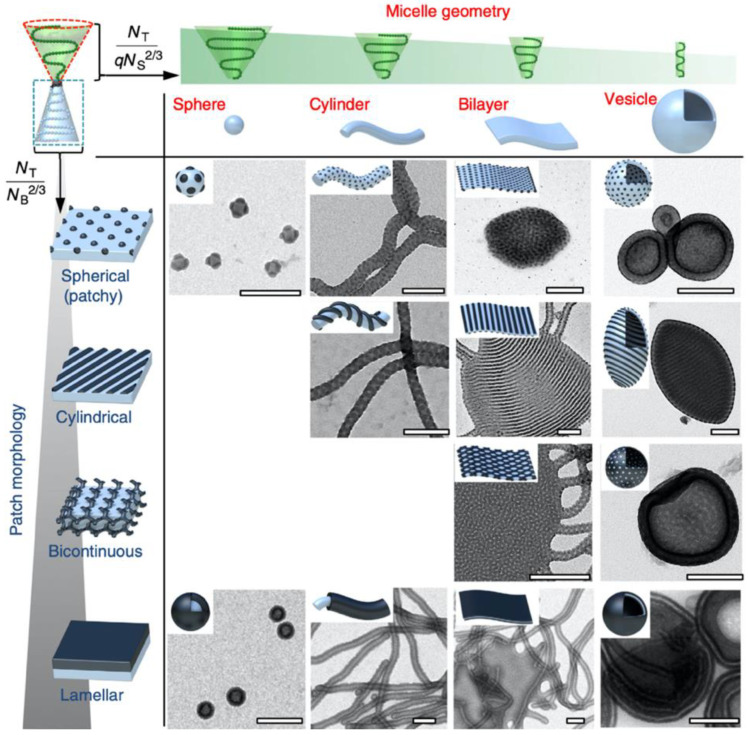
Multicompartment nanostructures from a series of PS-*b*-PB-*b*-PT triblock terpolymers. Reproduced with permission of [10]. Copyright 2016 Nature Publishing Group.

*Multicompartment particles:* As mentioned in the introduction, cross-linked microparticles (microgels or resins) have already been studied for some time as catalyst scaffolds. They can be modified in a facile manner and removed from the product by centrifugation or filtration. However, the potential of subdividing microparticles into multicompartment microparticles for the site-isolated storage of catalysts has not been studied much. This is surprising because microparticle synthesis *via* emulsion polymerization techniques are not only versatile with respect to size control, tunable particle swelling, and particle shapes, but also widely used in industry already. The simplest case of a core-shell microparticle can accommodate a catalyst in the core and shell, and led to an immediately applicable cascade catalyst [167]. Similarly, multicompartment micelles (MCMs) could offer a large variety of possibilities to store multiple catalysts in polymer compartments. MCMs typically contain two or more chemically different core compartments and have been reported frequently for ABC triblock terpolymers [21]. MCMs are not only synthesized from many different monomers, but can be controlled in shape, size, and compartment distribution. In a recent work, parameters for the predictive self-assembly of defined MCMs were investigated to create a combinatorial library of micelle and compartment shapes (Figure 20) [10]. Core-shell-corona micelles can be seen as the simplest MCM and have already been successfully employed for cascade catalysis with up to three catalytic steps (Section 4). The main challenge is to introduce multiple catalytic sites into compartments without catalyst interference in their activated state. If successful, this concept rewards with catalyst systems, where active species are located in few nanometer distance. Compartment sizes are typically in the range of 10–50 nm, which corresponds to the diffusion path from catalytic center to center. Beyond installing catalysts, MCMs could also be equipped with other functionalities such as reservoir compartments for product storage to free space around the catalytic center [109] or as sacrificial compartment for disassembly into micelle fragments to release the product.

*Biopolymer scaffolds:* In nature, cells are able to exploit compartmentalization to perform sequential enzyme-catalyzed processes. One of the main issues in constructing a synthetic artificial system mimicking these cellular processes is the increasing compatibility problem when biocatalysts are combined with other chemocatalysts. A viable method for catalyst site isolation apart from the previous mentioned polymeric support approaches are artificial metalloenzymes that use biopolymeric materials for catalyst implementation and separation [168]. In an effort to combine the virtues of enzymes and organic metal catalysts, a cascade system was designed involving the transformation of an organometallic catalyst into an artificial metalloenzyme *via* host-guest chemistry without interference with enzymes [169]. For instance, a metalorganic iridium complex was covalently attached to biotin and bound to streptavidin to form an artificial transfer hydrogenase (ATHase) preventing catalytic inactivation with other natural enzymes. The ATHase was combined with a variety of redox enzymes and cofactors to perform reaction cascades such as reduction/deracemization of prochiral imines to cyclic amines, stereoinversion of natural nicotine to a chiral alcohol, or the conversion of pipecolic acid to lysine. This example underlined the viability of biocatalytic cascade reactions by exploiting the incorporation of an organometallic catalyst into a protein scaffold without association with other enzymes. DNA origami is another tool that allows the precise formation of scaffolds with specific binding locations for the site isolation of catalysts. A biocatalytic cascade system was reported using nanoreactors assembled from tubular DNA origami units [170]. Initially, both DNA origami building blocks were constructed separately from different DNA strands to host different enzymes. Each building block was modified with neutravidins (NTVs) to facilitate the binding of the biocatalysts. GOx and HRP were then modified with biotin and separately anchored to avidin on the two origami scaffolds. The origami units could be linked together *via* DNA base pairing and formed a robust tubular nanoreactor. Glucose and 3,3′,5,5′-tetramethylbenzidine (TMB) were added as substrates, whereas the first was catalyzed by GOx under the release of H_2_O_2_, which was consumed in the HRP-catalyzed oxidation of TMB to TMB*. In light of the extraordinary complexity of today’s DNA origamis and the molecular precision with which functional moieties can be placed in 1D, 2D, and 3D [171], their combination with two or more site-isolated catalysts could be the foundation for more complex cascade reactions.

**Figure 21:**
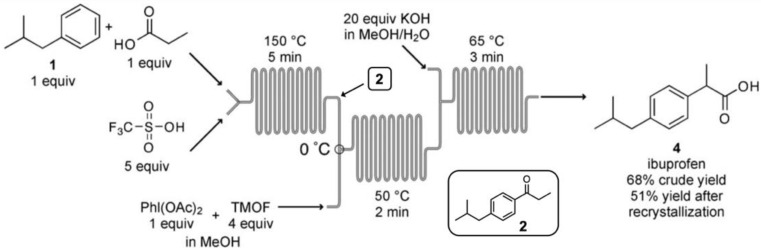
Continuous flow synthesis of ibuprofen. Reproduced with permission of [173]. Copyright 2009 Wiley-VCH.

*Scale-up through Continuous-Flow Synthesis:* Although fundamental research is absolutely necessary to foster our understanding of underlying processes and mechanisms, at some point, their feasibility for technological applications needs to be part of the design strategy. The above-described nanoreactors are mostly colloidal additives that are certainly advantageous due to facile catalyst removal or the possibility to conduct catalysis in specific environments. Another step along the road to technological application is to transfer multistep reactions from discontinuous batch reactors to continuous flow reactors for facile upscaling of products [172]. Continuous flow reactions benefit from high surface area to volume ratio as well as rapid heat transfer and mixing. One of the challenges of continuous flow reactors (or microreactors) is that every reaction step needs to be compatible with excess reactants and by-products, which may require the redesign of reaction pathways. An example of a multi-step reaction in continuous flow was demonstrated for the synthesis of ibuprofen [173]. After evaluation of several reactants and synthesis parameters, the setup produced 9 mg min^−1^ of crude ibuprofen in a three-step process that did not require purification of the intermediates (Figure 21). The first step was a Friedel-Crafts alkylation of isobutylbenzene with propionic acid in the presence of triflic acid providing 2 in 90% yield at 150°C in 5 min at a flowrate of 87.5 µL min^−1^. The subsequent 1,2-aryl migration to 3 in 70% yield was realized in 2 min at 0 °C by addition of trimethyl orthoformate and iodobenzene diacetate in methanol (excess triflic acid of the first step participated also in the second step). Cooling and mixing of the reactants were realized by glass beads within the flow reactor. To obtain the final product, 3 was hydrolyzed with KOH at 65 °C for 3 min.

*Automation.* Global network, data mining, and deep learning algorithms provide further opportunities in the future to harness the power of multiple laboratories for the synthesis of complex pharmaceuticals irrespective of the location. Remote control of automated systems brings a whole new set of challenges revolving around network security, security for the control systems, chemical supplies, online measurement of products, and emergency shut down procedures. Laboratories in Tokyo and Cambridge were coordinated *via* a cloud server in Los Angeles to synthesize the pharmaceuticals tramadol, lidocaine, and bupropion [174]. The operator in Los Angeles steered the automated instruments to screen reaction conditions and optimize conversion, consumption of starting material, and material throughput. Exemplified on tramadol, the Grignard reaction of (3-methoxy)phenylmagnesium bromide with 2-((dimethylamino)methyl) cyclohexanone was conducted in a flow reactor and the conversion monitored with a FlowIR unit. After screening residence time, temperature, and ratio of ketone to Grignard in 9 reactions over the course of 3 h, tramadol was obtained in 86% yield with 10.9 min residence time at 41 °C and 1.6 equiv. of Grignard. This optimization clearly demonstrated the potential to operate automated systems for chemical synthesis across long distances, which could be useful to increase safety when handling hazardous reactants, aid in the synthesis of complex chemical in remote locations, or to improve quality of live (coincidentally, this review was written during a global pandemic and remote controlled reactors could have compensated for restricted access to laboratories). Automated processes paired with artificial intelligence may allow the fast and coordinated design of synthetic pathways for more efficient production of complex pharmaceuticals or rapidly screen for entirely new cascade reaction routes.

## 7. Conclusions

Catalysis has such a great impact on all fields of science that advancements occur on a daily basis. We intended to summarize some examples from a personal perspective that highlight the great potential of combining catalysts with polymer compartments into multifunctional nanoreactors. Although in recent years, tremendous progress has been made in compartment synthesis, incorporation of catalysts, and the spatial separation of mutual incompatible catalysts in close proximity, there are still several challenges that need to be overcome regarding applications. Today, conventional catalytic systems still dominate industry that specializes on mass production and high throughput, because reaction vessels, controlled flow systems, and catalyst activities are highly optimized. Exciting opportunities arise in the development of advanced polymer pockets with respect to the above-mentioned perspectives which may lead to the incorporation of polymer-based nanoreactors in specialized industrial branches, when effective scale-up and high compatibility with different environments (cells, reactors, electronics) are resolved. Polymer-supported catalysts may find application in the production of fine chemicals and pharmaceuticals where the reduction of multi-step reactions to one-pot reactions promises great benefits, e.g., the reduction of solvent waste, reaction time, and energy. The proof of concept for efficient polymer compartment catalysis is there, which is bound to broaden their technical realization. With the increasing desire to reduce multistep reactions to as few steps as possible, the protection of catalysts in complex multicomponent mixtures will become progressively more important in the near future.
